# Multiscale modeling uncovers 7q11.23 copy number variation–dependent changes in ribosomal biogenesis and neuronal maturation and excitability

**DOI:** 10.1172/JCI168982

**Published:** 2024-07-15

**Authors:** Marija Mihailovich, Pierre-Luc Germain, Reinald Shyti, Davide Pozzi, Roberta Noberini, Yansheng Liu, Davide Aprile, Erika Tenderini, Flavia Troglio, Sebastiano Trattaro, Sonia Fabris, Ummi Ciptasari, Marco Tullio Rigoli, Nicolò Caporale, Giuseppe D’Agostino, Filippo Mirabella, Alessandro Vitriolo, Daniele Capocefalo, Adrianos Skaros, Agnese Virginia Franchini, Sara Ricciardi, Ida Biunno, Antonino Neri, Nael Nadif Kasri, Tiziana Bonaldi, Rudolf Aebersold, Michela Matteoli, Giuseppe Testa

**Affiliations:** 1 European Institute of Oncology (IEO) IRCCS, Milan, Italy.; 2 Human Technopole, Milan, Italy.; 3 Computational Neurogenomics, D-HEST Institute for Neuroscience, Federal Institute of Technology (ETH) Zürich, Zürich, Switzerland.; 4 Department of Biomedical Sciences, Humanitas University, Milan, Italy.; 5 IRCCS Humanitas Research Hospital, Milan, Italy.; 6 Department of Biology, Institute of Molecular Systems Biology, ETH Zürich, Zürich, Switzerland.; 7 Department of Oncology and Hemato-Oncology, University of Milan, Milan, Italy.; 8 Hematology Unit, Fondazione IRCCS Ca’ Granda Ospedale Maggiore Policlinico, Milan, Italy.; 9 Department of Cognitive Neurosciences, RadboudUmc, Donders Institute for Brain Cognition and Behaviour, Nijmegen, Netherlands.; 10 Department of Biosciences, University of Milan, Milan, Italy.; 11 National Institute of Molecular Genetics, Fondazione Romeo ed Enrica Invernizzi, Milan, Italy.; 12 Integrated Systems Engineering Srl, c/o OpenZone, Bresso, Milan, Italy.

**Keywords:** Neuroscience, Stem cells, Neurodevelopment, Psychiatric diseases, Translation

## Abstract

Copy number variation (CNV) at 7q11.23 causes Williams-Beuren syndrome (WBS) and 7q microduplication syndrome (7Dup), neurodevelopmental disorders (NDDs) featuring intellectual disability accompanied by symmetrically opposite neurocognitive features. Although significant progress has been made in understanding the molecular mechanisms underlying 7q11.23-related pathophysiology, the propagation of CNV dosage across gene expression layers and their interplay remains elusive. Here we uncovered 7q11.23 dosage–dependent symmetrically opposite dynamics in neuronal differentiation and intrinsic excitability. By integrating transcriptomics, translatomics, and proteomics of patient-derived and isogenic induced neurons, we found that genes related to neuronal transmission follow 7q11.23 dosage and are transcriptionally controlled, while translational factors and ribosomal genes are posttranscriptionally buffered. Consistently, we found phosphorylated RPS6 (p-RPS6) downregulated in WBS and upregulated in 7Dup. Surprisingly, p-4EBP was changed in the opposite direction, reflecting dosage-specific changes in total 4EBP levels. This highlights different dosage-sensitive dyregulations of the mTOR pathway as well as distinct roles of p-RPS6 and p-4EBP during neurogenesis. Our work demonstrates the importance of multiscale disease modeling across molecular and functional layers, uncovers the pathophysiological relevance of ribosomal biogenesis in a paradigmatic pair of NDDs, and uncouples the roles of p-RPS6 and p-4EBP as mechanistically actionable relays in NDDs.

## Abstract 2

Copy number variations at 7q11.23 alter kinetics of neuronal maturation, ribosomal biogenesis, the mTOR pathway, and neuronal intrinsic excitability.

## Graphical abstract

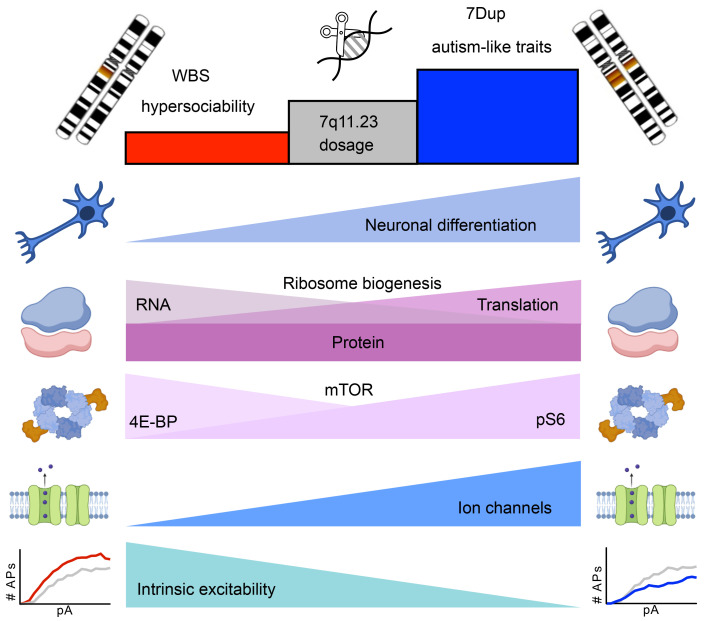

## Introduction

With over a thousand associated genes and an increasing number of polygenic risk variants, neurodevelopmental disorders (NDDs), in particular autism spectrum disorder (ASD) and intellectual disability (ID), continue to elude molecular understanding. Two key challenges for the field are how these converge into similar pathologies, and at which stage and scale it makes most sense to investigate them mechanistically.

Rare monogenic conditions, through their well-defined and amenable genetics, offer unique opportunities for exploring the reach of disease-modeling approaches and testing their potential for both mechanistic dissection and translational inroads. On the other hand, syndromes caused by symmetrically opposite copy number variation (CNV) are particularly informative since they offer the opportunity to identify dosage-dependent changes in specific molecular mechanisms (1). Within them, the 7q11.23 CNV leads to a pair of multisystemic syndromes with shared and opposite neurocognitive and behavioral profiles; the hemideletion causes Williams-Beuren syndrome (WBS; Online Mendelian Inheritance in Man [OMIM] 194050), while its hemiduplication causes 7q microduplication syndrome (7Dup; OMIM 609757). Even though both NDDs share some characteristics, such as mild to moderate intellectual disability, anxiety, attention-deficit/hyperactivity disorder (ADHD), and facial dysmorphic features, they also differ in others (2, 3); while WBS is characterized by deficits in visuospatial construction and relative strength in language and hypersociability (3), 7Dup is associated with speech delay and ASD (2). The strikingly opposite patterns in these complex cognitive features of the 2 conditions suggest that the symmetry is maintained all the way from the original CNV through the various layers of biological organization and regulation, up to behavior and cognition, while the presence of shared features implies that some of the molecular underpinnings might be similar between the 2 conditions. The 7q11.23 locus comprises 26–28 genes, including several key regulators of transcription and translation. We previously dissected 7q11.23-related transcriptional dysregulation at the induced pluripotent stem cell (iPSC) stage, which was then selectively amplified upon the onset of neuronal differentiation (4). In addition, by using cortical brain organoids as a model, we recently found dosage-dependent impaired dynamics of neural progenitor proliferation, transcriptional imbalances, and a precocious production of excitatory neurons in 7Dup, which was rescued by restoring physiological levels of a key transcription factor (TF) of the 7q11.23 region, GTF2I (5). To transit properly in functional neurons, neural stem cells must integrate complex external and internal signals to divide, differentiate, migrate, and mature properly. All these stages are guided by extrinsic factors that promote timely changes in transcription programs driving the complex process of neurogenesis. Many of these external and internal signals converge on the mammalian target of rapamycin (mTOR) pathway, which is a master regulator of cell growth, proliferation, metabolism, and protein translation. There is a body of evidence regarding the role of mTOR in neural stem cell differentiation, neural progenitor cell (NPC) migration, dendrite development, and neuronal maturation and function (6, 7). Regulation of these processes is ascribed to the 2 primary downstream effectors of mTOR, phosphorylated 4E-binding protein 1 and 2 (p-4EBP1 and -2) and phosphorylated p70 S6 kinase 1 and 2 (p-S6K1 and -2) along with its target phosphorylated ribosomal protein S6 (p-RPS6). However, even though these 2 proteins are often treated as equivalent and p-RPS6 is used as the sole readout of mTOR activity, growing evidence demonstrates their distinct biological functions (8, 9). In line with the role of the mTOR pathway, aberrant mTOR activity is associated with numerous neuropsychiatric conditions, including NDDs, ASD, and ID (10). Similarly, ribosome biogenesis has also recently emerged as a possible underlying mechanism linking various NDDs (11), thus providing further support to the idea that mutations strongly associated with NDDs affect all layers of gene expression.

Despite the significant contributions that we and several other teams made to the understanding of the molecular mechanisms underlying 7q11.23-related pathophysiologies (4, 5, 12–18), the interplay between different layers of gene expression shaped by 7q11.23 CNV remains elusive. We hypothesized that the symmetry between 7q11.23 CNVs can act as a uniquely informative conduit in deciphering clinically relevant pathways underlying sociability and language competence. By integrating transcriptomics, translatomics, proteomics, and electrophysiological analysis of glutamatergic neurons derived from 7q11.23 neurotypical and CNV patients, along with a fully isogenic allelic series that recapitulates the dosages of the 7q11.23 locus, we were able to uncover the mechanisms that link 7q11.23 genetic dosage imbalances to key NDD phenotypes.

## Results

### Generation of an isogenic allelic series of 7q11.23 CNVs.

**Figure 1:**
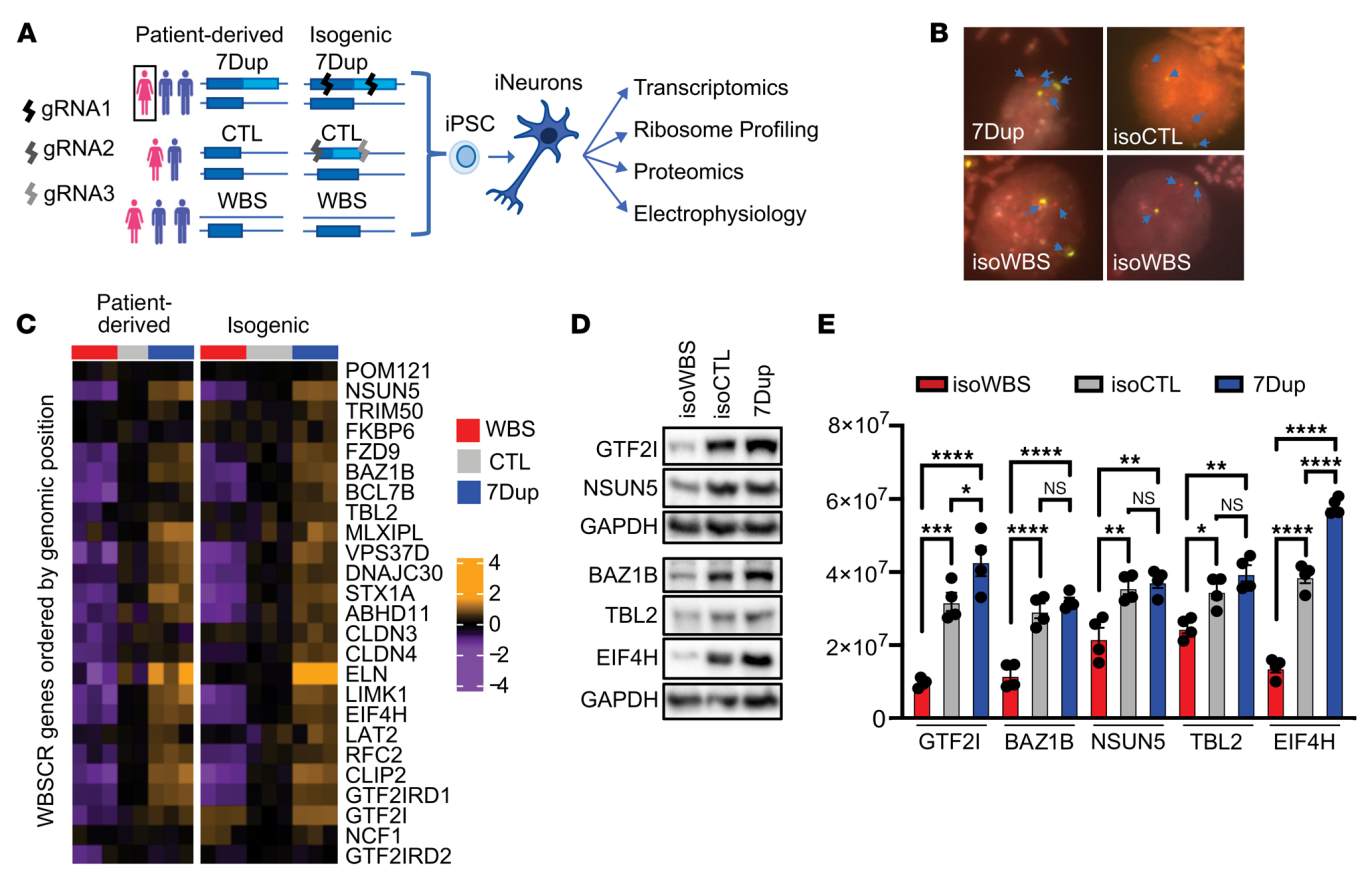
7q11.23 isogenic iNeurons preserve dosage. (**A**) Scheme of experimental design and generation of 7q11.23 isogenic lines. (**B**) Two-color FISH analyses using 7 alpha satellite probes (see Supplemental Methods) as a control for the chromosomal number (yellow) and *ELN*, a WBSCR gene (red). *ELN* showed 3 signals in 7Dup, 2 in isoCTL, and 1 in isoWBS, corresponding to the 7q11.23 copy number in respective clones. (**C**–**E**) WBSCR genes maintain the dosage at the RNA and protein levels. RNA-seq data for WBSCR genes are shown for both patient-derived and isogenic neurons for all 3 genotypes. Although *GTF2I* transcripts were not downregulated in isoWBS in the RNA-seq analysis, both the translatome and proteome data showed 7q11.23 dosage–dependent expression (Supplemental Figure 1, F and G) that was also confirmed by Western blot (Figure 1D and Supplemental Figure 1H), suggesting that the upregulation observed at the mRNA level is probably an artifact of sequencing of repetitive regions. Western blot results from the same neuronal preparation, run on 2 gels, are shown in **D**. GAPDH was used as a loading control. Quantification of Western blots (shown in **D** and Supplemental Figure 1H) is shown as relative expression in **E**. Non-normalized data are shown as mean ± SEM (*n* = 4). The statistical comparisons were done with 1-way ANOVA followed by Tukey’s multiple-comparison test. *P < 0.05; **P < 0.01; ***P < 0.001; ****P < 0.0001.

Human genetic heterogeneity poses a formidable challenge for disease modeling, being at once the very aspect that one would wish to capture for patient-tailored precision, but also a significant potential confounder for the dissection of disease mechanisms. Isogenic and patient-derived settings can thus provide complementary insights, allowing to focus with the former on the phenotypes exclusively imputable to the mutation at hand, while excluding with the latter any potential artifact arising from the genetic manipulation per se or from the spurious interaction of the mutation with the given genetic background. Building on the empirical benchmarks we had previously derived from the meta-analysis of the 2 large iPSC resources, we set out to complement our cohort of patient-derived iPSC lines (4, 19) with a fully isogenic allelic series that recapitulates, in the same genetic background, the 3 dosages of the 7q11.23 interval (hemiduplicated, control [CTL], and hemideleted; Figure 1A). To this end, we exploited the presence of the 7q11.23 duplication in the cells originating from a 7Dup patient and targeted the Cas9 onto the WBS critical region (WBSCR). We used a single guide RNA (gRNA) that simultaneously recognized both duplicated sequences and consequently introduced an intrachromosomal deletion, thus effectively generating an isogenic healthy control (isoCTL) in a female 7Dup background (Figure 1A). Successively, we performed a second round of CRISPR/Cas9 editing starting from isoCTL to generate an isogenic WBS (isoWBS) line, using 2 gRNAs that flank the whole WBSCR (Figure 1A). The deletions were screened by digital PCR assays on genomic DNA and validated by FISH analysis (Figure 1B and Supplemental Figure 1A; supplemental material available online with this article; https://doi.org/10.1172/JCI168982DS1). Western blot analysis confirmed that isogenic iPSC lines preserved a 7q11.23 dosage imbalance of proteins encoded by genes located in the WBSCR interval (Supplemental Figure 1, B and C), while a short tandem repeat (STR) analysis confirmed their identity (Supplemental Figure 1D). Next, we generated neurogenin 2–driven (*Ngn2*-driven) induced cortical glutamatergic neurons (iNeurons) (19) by ectopic expression of *Ngn2* delivered with a PiggyBac transposon system (19, 20), which ensured high reproducibility between different rounds of differentiation (Supplemental Figure 1E). Isogenic iNeurons faithfully recapitulated the dosage imbalances of WBSCR genes, similar to those of patient-derived iNeurons, at the level of both transcriptome and proteome (Figure 1, C–E, and Supplemental Figure 1, F–H).

Correlation analysis of transcriptome signatures from 30-day-old patient-derived and isogenic iNeurons with the signature of the human developing neocortex (21) revealed that our differentiation paradigm recapitulates cortical early upper layer neurons (gestation week [GW] 16–18; Supplemental Figure 2A), in line with previous reports (22–24). We further performed KaryoStat analysis (see Supplemental Methods) to assess the genomic integrity of neurons derived from isogenic lines. The analysis uncovered a large amplification of chromosome 14 (Chr14) in isogenic lines, which probably originated in the mosaicism of the original patient line used for the generation of the isogenic lines (Supplemental Figure 2, B and C; the list of amplified genes is provided in Supplemental Table 1). In line with the Chr14 amplification in all 3 genotypes, the comparison of the transcriptomes did not show any substantial change in the expression of the amplified region between the original 7Dup line and the isogenic derivatives (Supplemental Figure 2D). Furthermore, we inspected for possible interactions between proteins encoded by genes located at Chr14 and those within the WBSCR (Supplemental Figure 2, E and F). While a handful of Chr14 proteins interacting with WBSCR genes showed small variations in expression, with an absolute log(fold change) (|logFC|) of 0.457 or less (Supplemental Figure 2F), we see no indication of a significant impact of Chr14 trisomy on the described 7q11.23 phenotypes.

Therefore, we concluded that this unique series of isogenic lines offers the opportunity to study the effect of 7q11.23 CNV and reveal disease-relevant endophenotypes in a highly controlled setting and in conjunction with patient-derived lines.

### 7q11.23 dosage alters neuronal differentiation in a symmetrically opposite manner.

**Figure 2:**
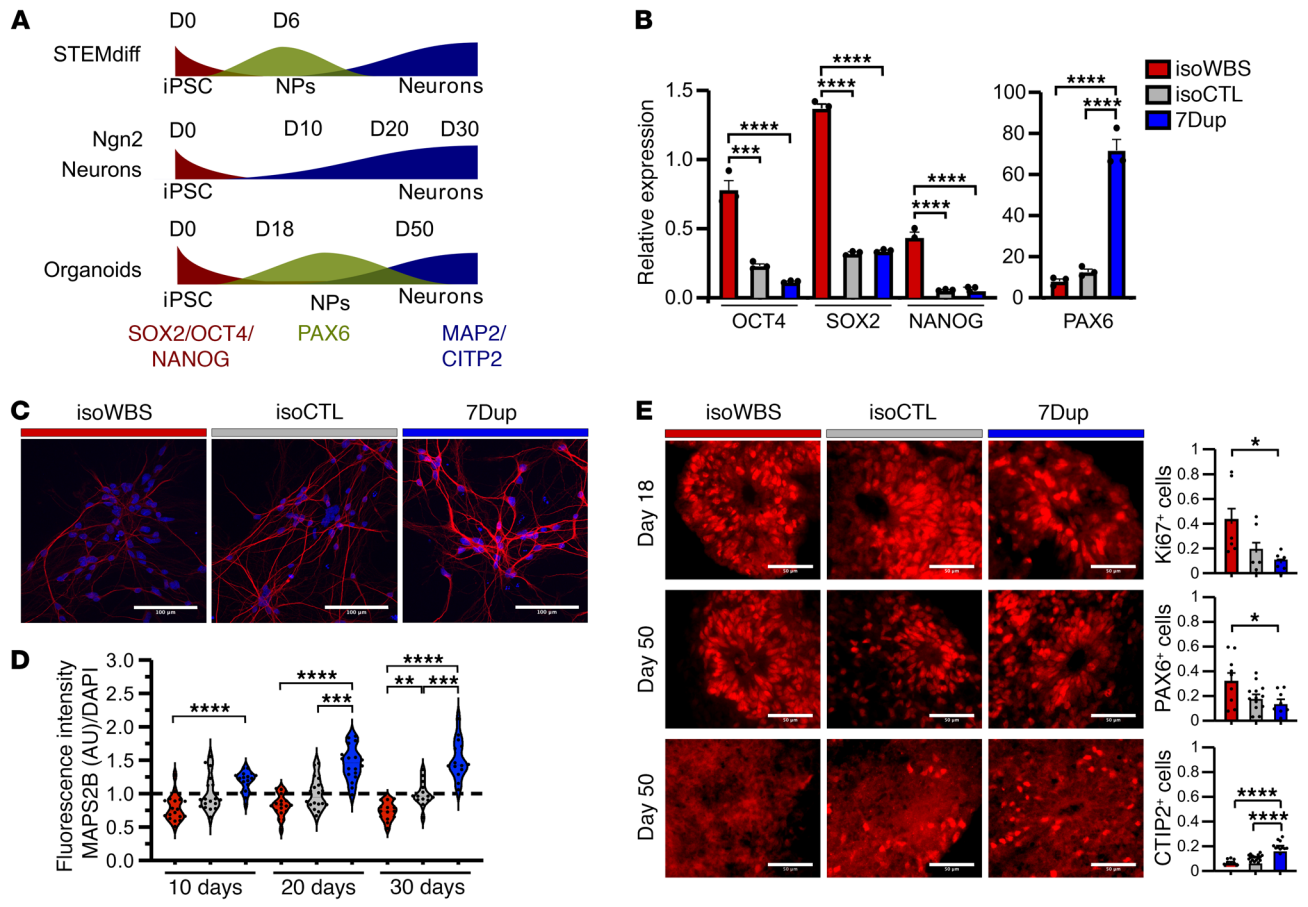
7q11.23 hemideletion delays, whereas hemiduplication accelerates, neuronal differentiation. (**A**) Diagram showing the timing of neuronal differentiation in 3 different neuronal models: STEMdiff-driven (dual-Smad-based; see Supplemental Methods) and *Ngn2*-driven iNeurons, and cortical brain organoids. The expected change in profiled markers in each model is schematized. Red, iPSCs; green, NPCs; blue, neurons. (**B**) Expression of stem markers (*OCT4*, *SOX2*, and *NANOG*) and NPC marker *PAX6* in early NPCs (*n* = 3) measured by qPCR. (**C**) Representative immunofluorescence images of 30-day-old *Ngn2*-iNeurons from isoWBS, isoCTL, and 7Dup stained for the mature neuronal marker MAP2B (red) and with DAPI (blue). Scale bars: 100 μm. (**D**) Quantification of MAP2B fluorescence intensity versus the cell number in *Ngn2*-iNeurons assessed at 10, 20, and 30 days of 2 independent differentiations (14–18 fields of view). IsoWBS and 7Dup were normalized to controls. (**E**) Immunofluorescence in cryosections of cortical organoids from isogenic lines on days 18 and 50 for proliferative marker Ki67, neural progenitor marker PAX6, and neuronal postmitotic marker CTIP2. Scale bars: 50 μm. First row, quantification of Ki67: isoWBS *n* = 3 organoids, isoCTL *n* = 4, 7Dup *n* = 3; second row, quantification of PAX6: isoWBS *n* = 5 organoids, isoCTL *n* = 4, 7Dup *n* = 3; third row, quantification of CTIP2: isoWBS *n* = 5 organoids, isoCTL *n* = 4, 7Dup *n* = 3. Data points are organoids’ sections from 2 independent experiments. All data are shown as mean ± SEM. The statistical comparisons were done with 1-way ANOVA followed by Tukey’s multiple-comparison test. *P < 0.05; **P < 0.01; ***P < 0.001; ****P < 0.0001.

Upon differentiation into iNeurons, the occurrence of dosage-dependent differences in the dynamics of morphological changes (Supplemental Figure 2G) prompted us to systematically compare the kinetics of differentiation between genotypes, starting from the earliest stages. We exploited 3 different neuronal models (aligned timelines of specific cell types and the expression of the analyzed markers in each model are depicted in Figure 2A) and assessed the expression of (i) pluripotency (*OCT4*, *SOX2*, and *NANOG*) and neural progenitor (*PAX6*) markers in early NPCs differentiated with STEMdiff (day 5; Figure 2B); (ii) mature neuron marker MAP2B in *Ngn2*-iNeurons (day 10, 20, and 30; Figure 2, C and D); and (iii) proliferative marker Ki67, PAX6, and postmitotic deep-layer neuron marker CTIP2 on day 18 and 50, respectively, in brain organoids (Figure 2E). In all 3 neuronal models, we found symmetrically opposite kinetics of differentiation (by symmetrically opposite, we mean that WBS and 7Dup go in opposite directions compared with CTL). Thus, while isoWBS NPCs still expressed pluripotency markers (*OCT4*, *SOX2*, and *NANOG*), 7Dup already had high levels of the neural progenitor marker *PAX6* on day 5 of differentiation, indicating that isoWBS had delayed and 7Dup had accelerated differentiation kinetics compared with isoCTL (Figure 2B). The expression of MAP2B followed 7q11.23 dosage. On day 10 of differentiation, there was a statistically significant difference only between isoWBS and 7Dup (*P* < 0.0001), on day 20 also between isoCTL and 7Dup (*P* < 0.001), while on day 30, in addition to isoWBS versus 7Dup and isoCTL versus 7Dup, we found a significant difference also between isoWBS and isoCTL (*P* < 0.01), which suggests that with time the symmetrically opposite kinetics of differentiation becomes more apparent in this model (Figure 2, C and D). Similarly to iNeurons, also in brain organoids symmetrically opposite kinetics of differentiation became more apparent at later time points. Ki67 was enriched in isoWBS compared with 7Dup on day 18 (*P* < 0.05; Figure 2E), while on day 50, when brain organoids contain a mixed population of NPCs and postmitotic neurons (25), PAX6 was higher in isoWBS compared with 7Dup (*P* < 0.05), whereas CTIP2 was enriched in 7Dup compared with both isoCTL and isoWBS (both *P* < 0.0001), confirming symmetrically opposite kinetics of differentiation (Figure 2E). These results are in agreement with the longitudinal analysis of brain organoids and *Gtf2i* dosage–specific murine models in our recent paper (5), where we also found symmetrically opposite dynamics of neural progenitor proliferation and accelerated production of excitatory neurons in 7Dup. Therefore, these data robustly underscore the idea that 7q11.23 gene dosage imbalances regulate the timing of neuronal differentiation, both across models and in patient-specific versus isogenic designs.

### Symmetrically opposite transcriptional regulation of translation and neuronal transmission genes in WBS and 7Dup.

**Figure 3:**
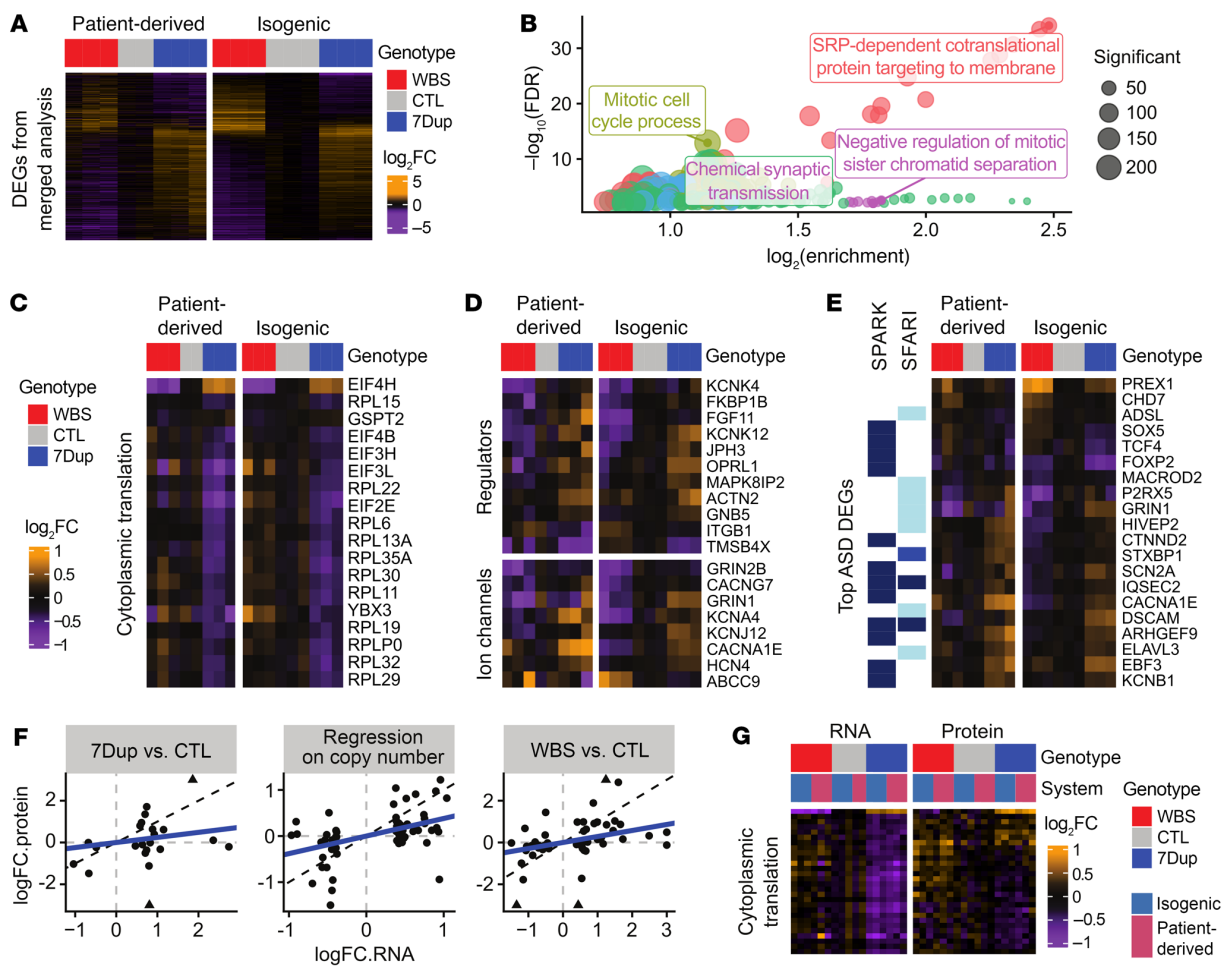
Robust transcriptional changes in translation- and neural transmission–related genes. (**A**) Fold changes of DEGs in the merged analysis of isogenic and patient-derived lines (in either WBS vs. CTL, 7Dup vs. CTL, or regression on 7q11.23 copy number), showing robust transcriptional signatures that are largely symmetrically opposite between genotypes. SRP, signal recognition particle. (**B**) Enriched GO terms in the regression on 7q11.23 copy number. Similar terms are clustered (denoted by colors) and only the top term per cluster is shown. (**C**–**E**) Top DEGs associated with translation (**C**), ion channels and their regulation (**D**), or ASD (**E**). (**F**) Comparison of fold changes at the RNA and protein levels (for the union of genes found significant at either level), in each of the 3 comparisons performed. (**G**) Expression of the transcriptionally dysregulated translation genes that could also be measured at the proteome level, highlighting a buffering in 7Dup.

Isogenic and patient-derived iNeurons showed remarkably consistent 7q11.23-associated transcriptome changes (Supplemental Figure 3A; results obtained in the 2 models are shown in Supplemental Table 2), though — as expected — not all changes were significant in both systems, highlighting the value of using the 2 modeling paradigms in their complementarity. A merged analysis of the data sets revealed a highly reproducible signature of the CNV across 2132 differentially expressed genes (DEGs; either in WBS vs. CTL, 7Dup vs. CTL, or in the regression on copy numbers), including 1061 high-confidence genes (FDR < 0.01 and changing by at least 40%), which formed the basis of our downstream analysis (Figure 3A). This revealed a largely linear dosage sensitivity, with 85% of genes having a fold change in opposite directions in WBS and 7Dup (although not always with the same magnitude, and generally slightly weaker in 7Dup; see Supplemental Figure 3B), in line with the partially symmetrically opposite phenotypes of the syndromes in the neural domain. The 7q11.23-sensitive genes showed highly significant enrichments for several biological processes, with ribosomal genes and translation initiation factors being downregulated when 7q11.23 copy number dosage was increased (Figure 3B in red, and Figure 3C), whereas ion channels and synaptic transmission genes were upregulated (Figure 3B in green, and Figure 3D), all changes in alignment with aforementioned symmetrically altered kinetics of differentiation (26, 27). Enrichment analysis for genotype-specific DEGs was less clearly related to neuronal function (Supplemental Figure 3, C and D), further suggesting the relevance of symmetrically opposite changes for the neural domain phenotypes. In addition, the enrichments for cell cycle–related terms in WBS (Supplemental Figure 3C) are in agreement with the observed differences in neuronal differentiation and increased proliferation of NPCs in isoWBS organoids (Figure 2E and ref. 5). Finally, the DEGs were significantly enriched for ASD-associated genes (Fisher’s *P* = 1 × 10^–12^; the most significant ASD-associated DEGs are shown in Figure 3E), underscoring the impact of 7q11.23 dosage on critical genes associated with sociability and cognitive phenotypes.

### Neuronal transmission genes are transcriptionally controlled, while translation-related genes show dosage-dependent posttranscriptional regulation.

As expected from the higher measurement noise and lower coverage of proteomics (2300 unique proteins quantified across all samples, and 3057 quantified across at least 75% of the samples), only 27% of DEG RNAs could be measured also at the protein level. Although very few of those DEGs passed multiple testing in the proteome, the relative changes in the proteome were largely correlated with the transcriptome, albeit with partial buffering (Figure 3F, i.e., mitigation of the impact of mRNA alterations on the proteome; refs. 28, 29). Although proteins forming complexes often show stronger buffering, we observed only a weak effect in this direction (Supplemental Figure 3F). Of note, most of the buffering appeared condition specific, like in the case of ribosomal protein and translation initiation factor buffering (Figure 3G).

**Figure 4:**
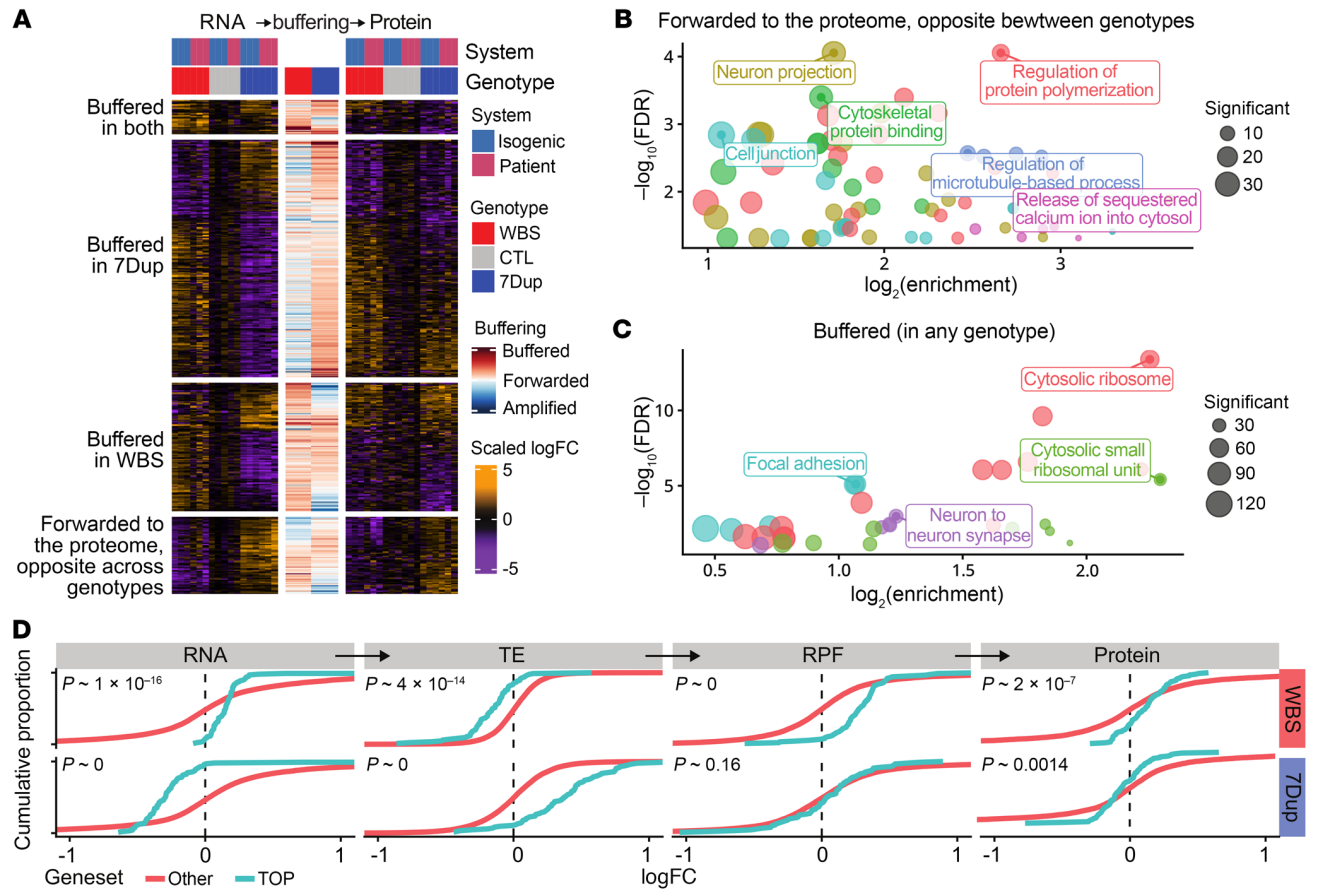
Posttranscriptional regulation. (**A**) Cross-layer clustering of DEGs reveals distinct patterns of transcriptional and translational regulation, emphasizing condition-specific buffering and genes forwarded to the proteome. Buffering coefficients (capturing the reduction or amplification of the fold change at the protein level) for each condition are shown in the center. (**B** and **C**) Enriched GO terms in the forwarded (**B**) and buffered (**C**) clusters. (**D**) Translational buffering opposes the transcriptional dysregulation of genes encoding TOP mRNAs in 7Dup. Cumulative distribution plots comparing the fold changes in both conditions and across gene expression layers of genes encoding 5′ TOP mRNAs to that of other genes are shown. RPF, ribosome-protected fragments; TE, translation efficiency.

To investigate the posttranscriptional regulation underlying the observed buffering, we performed analysis of ribosome-protected fragments (RPFs) in isogenic iNeurons. Integrated analysis across the 3 layers (transcriptome, translatome, and proteome) revealed significant buffering at the level of translation for a subset of genes and confirmed that these were distinct in WBS and 7Dup (Supplemental Figure 3, G and H). To explore these different sets of genes, we clustered the union of DEGs with significant differences at both RNA and protein levels, according to the direction of their fold change (with |logFC| < 0.2 considered 0) in each condition and layer (Figure 4A and Supplemental Table 3). Genes whose expression was roughly linearly correlated with copy number both at the RNA and protein levels (“forwarded opposite,” i.e., their symmetrically opposite dysregulation is forwarded from the transcriptome to the proteome) were related to protein polymerization, neuronal projections, synaptic plasticity, and ion transport (Figure 4B; some example genes are shown in Supplemental Figure 3E). For those genes, buffering was minor (regression of the protein logFC on RNA logFC yielded a slope of 0.8). In contrast, genes that were buffered at the protein level in either genotype were related to translation, mostly ribosomal proteins and translation initiation factors (Figure 4C and Supplemental Table 3). Other gene clusters did not show statistically significant enrichments. Hence, while the expression of genes most proximally related to neuronal transmission is primarily transcriptionally controlled, translation-related genes display a more complex, multilayered regulation that entails significant posttranscriptional buffering. Of note, many dysregulated translation-related genes belong to the group of 5′ terminal oligopyrimidine (TOP) mRNAs, which undergo coordinated translation control (30). We thus probed the fold change distribution (isoWBS vs. isoCTL and 7Dup vs. isoCTL) at the level of RNA, RPF, and proteins of the whole core set of genes with a 5′ TOP motif (31). While 5′ TOP genes tended to be transcriptionally downregulated in 7Dup and upregulated in WBS, we observed a highly significant (*P* < 2 × 10^–16^ by Kolmogorov-Smirnov test) opposite trend at the level of translation efficiency (TE), which buffered their expression at the protein level, pointing to a major translation remodeling counteracting transcriptional imbalances (Figure 4D).

### Genotype-specific mTOR dysregulation.

**Figure 5:**
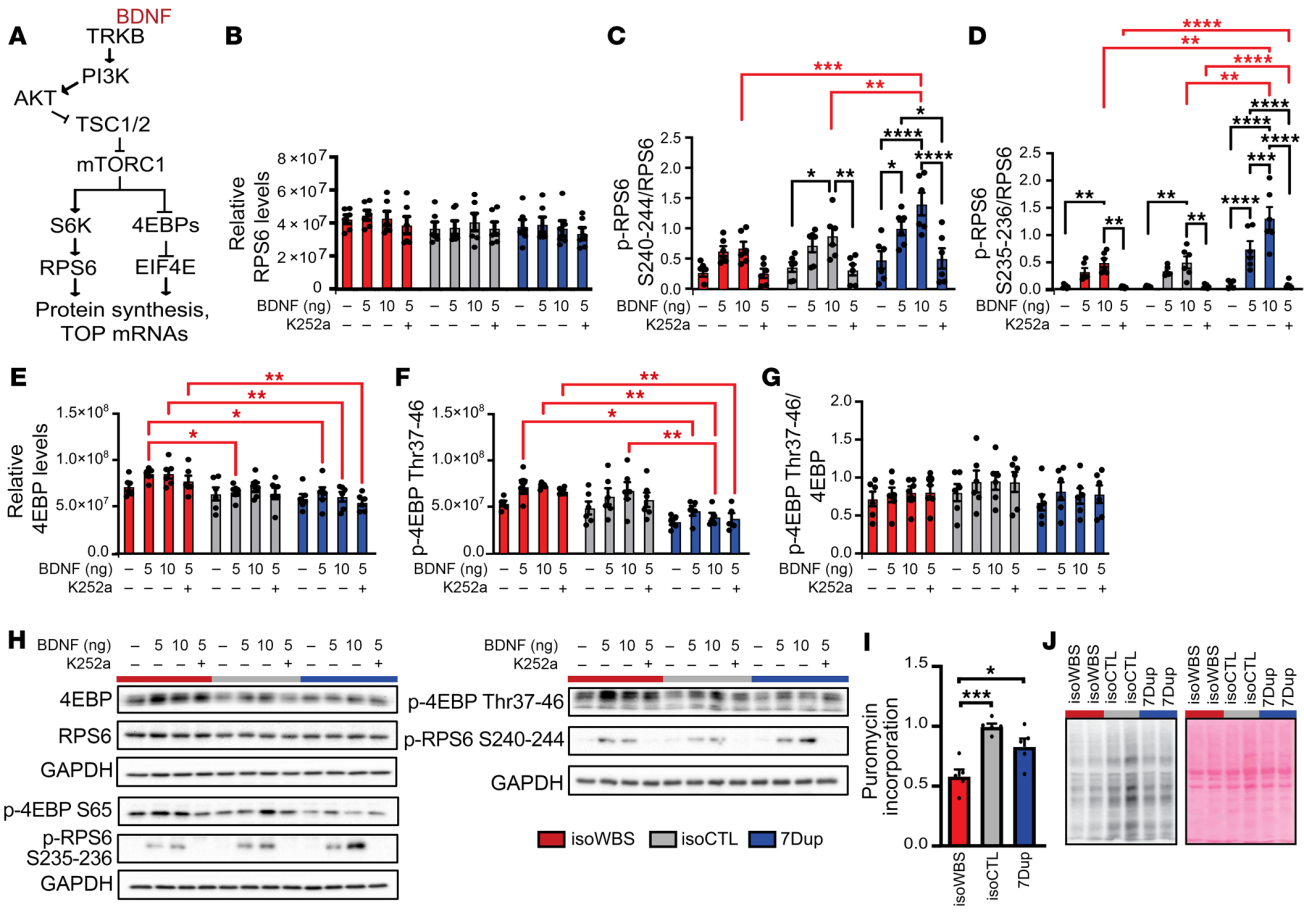
Genotype-specific dysregulation of p-RPS6, but not p-4EBP, in 30-day-old iNeurons. (**A**) Simplified scheme of mTOR signaling. (**B**–**G**) Quantification of Western blot analyses for total RPS6 (**B**), p-RPS6 S240/S244, and p-RPS6 S235/S236 normalized to RPS6 levels (**C** and **D** respectively), 4EBP (**E**), p-4EBP Thr37/Thr46 (**F**), and p-4EBP Thr37/Thr46 normalized to 4EBP levels (**G**). The experiment was done on 6 different iNeuron preparations, differentiated in 2 different rounds of differentiation. (**H**) Representative Western blot quantification for **B**–**G**. Other quantified blots are shown in Supplemental Figure 5. (**I** and **J**) Quantification of puromycin incorporation assay (**I**; *n* = 5) with representative Western blot (**J**). Other quantified blots are shown in Supplemental Figure 4G. All data are shown as mean ± SEM. The statistical comparisons were done with 2-way ANOVA followed by Tukey’s multiple-comparison test (**B**–**G**) or 1-way ANOVA followed by Tukey’s multiple-comparison test (**I**). *P < 0.05; **P < 0.01; ***P < 0.001; ****P < 0.0001. Black asterisks indicate significance between treatments, whereas the red asterisks indicate significance between genotypes.

The mTOR signaling pathway (Figure 5A) is the key regulator of the translation of 5′ TOP mRNAs (30). To assess its activity, we profiled the total protein levels and the phosphorylated forms of 2 principal downstream effectors of mTOR, p-RPS6 and p-4EBP. RPS6 can be phosphorylated at multiple sites, where phosphorylation at serine 240 and 244 (S240/S244) is specific for S6K1/2, whereas S235/S236 can be phosphorylated by multiple kinases, such as PKA, RSK, PKC, PKG, and DAPK, and S247 by CK1, in addition to S6K1/2 (8). Similarly, 4EBP1/2 can be phosphorylated at threonine 37 (Thr37), Thr46, S65, S70, S83, S101, and S112, where Thr37/Thr46 serve as a priming event and are specific for mTOR signaling (32). Since iNeurons are grown in a rich medium supplemented with various growth factors, we did not expect to see significant differences in the mTOR pathway activity in basal conditions. Thus, to see potential differences in the activity of the mTOR pathway, we treated iNeurons from the 3 genotypes with increasing amounts of brain-derived neurotrophic factor (BDNF), which activates the mTOR signaling pathway via the NTRK2 (TRKB) receptor, and with a TRKB inhibitor (K252a; ref. 33), on the fourth day following the last medium change (i.e., to ensure BDNF depletion). As expected, we did not observe any statistically significant changes in basal conditions, while the treatment highlighted differential activity of the mTOR pathway between genotypes (Figure 5 and Supplemental Figures 4 and 5). Consistent with translational buffering (Figure 4D), total RPS6 levels did not change between genotypes (Figure 5B), while treatment with increasing BDNF concentrations led to the progressive increase in phosphorylation levels at S240/S244 and S235/S236 and the corresponding decrease when pretreating cells with the TRKB inhibitor K252a (Figure 5, C and D). In concordance with increased translation of the TOP mRNAs specifically in 7Dup (Figure 4D), we observed more responsiveness in 7Dup to the BDNF treatment in comparison with isoWBS and isoCTL, which was true for both S240/S244 and S235/S236 (p-RPS6 at S240/S244 levels normalized to the total levels, Figure 5C; only p-RPS6 S240/S244 levels, Supplemental Figure 4, A and B; p-RPS6 at S235/S236 levels normalized to the total levels, Figure 5D; only p-RPS6 S235/S236 levels, Supplemental Figure 4, C and D). The genotype-specific effect was more enhanced at S235/S236 compared with S240/S244 (Figure 5, C and D; please consult the figures for the significance), indicating the contribution of more kinases in phosphorylating RPS6 downstream of TRKB. Surprisingly, p-4EBP responded instead to a much lesser extent to the BDNF treatment in all 3 genotypes, at both Thr37/Thr46 and S65 sites (Figure 5F and Supplemental Figure 4E). While we could observe the expected trend in the increase in phosphorylation upon BDNF treatment, none of the changes were statistically significant. However, we did observe statistically significant differences between genotypes in total 4EBP levels (Figure 5E; please consult the figure for the significance), which were reflected in the phosphorylated levels of 4EBP (Figure 5F for Thr37/Thr46 and Supplemental Figure 4E for S65). Consequently, as expected, when p-4EBP levels were normalized to the total protein levels, no statistically significant differences were observed for either of the phosphorylated forms of 4EBP (Figure 5G and Supplemental Figure 4F). Genotype-specific differences in the mTOR pathway (Figure 5 and Supplemental Figures 4 and 5) and transcriptional and translational dysregulation of the translation apparatus (Figure 4D) prompted us to verify global TE by puromycin incorporation assay. Consistent with decreased p-RPS6 levels upon activation with BDNF as well as higher total 4EBP levels in isoWBS when compared with 7Dup, we found reduced TE in isoWBS compared with 7Dup (Figure 5, I and J, and Supplemental Figure 4G; isoWBS vs. 7Dup, *P* < 0.05). Finally, we also found a significant reduction in TE in isoWBS when compared with isoCTL (Figure 5, I and J, and Supplemental Figure 4G; isoWBS vs. isoCTL, *P* < 0.001), suggesting that other components, in addition to the mTOR pathway, affect translation. In that regard, we checked the phosphorylation of EIF2α, but we did not observe any significant change (Supplemental Figure 4, H and I).

### Symmetrically opposite intrinsic excitability in WBS and 7Dup iNeurons.

**Figure 6:**
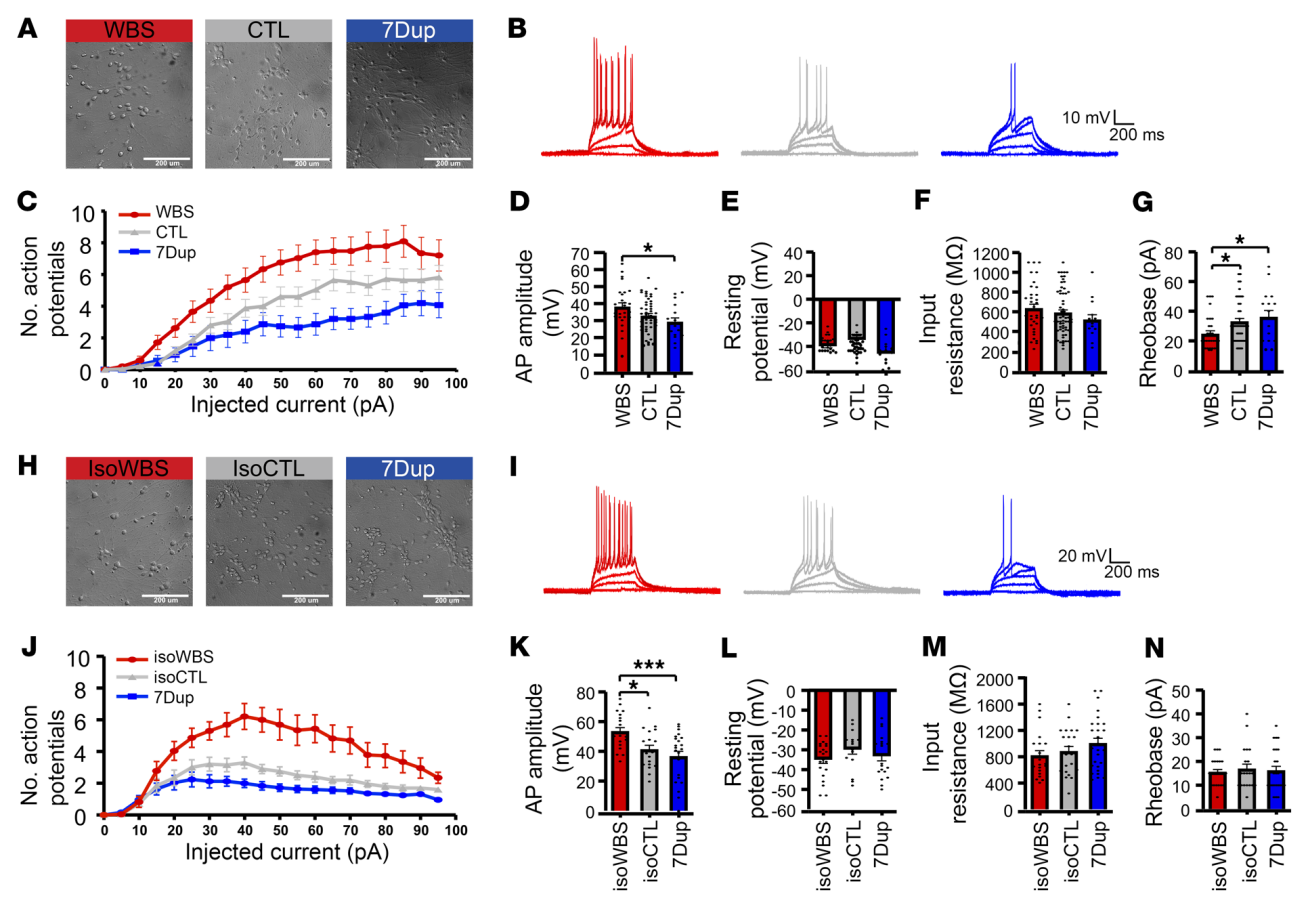
7q11.23 CNV causes symmetrically opposite neuronal excitability dynamics. (**A**) Bright-field images of CTL, WBS, and 7Dup patient-derived iNeurons. Scale bars: 200 μm. (**B**) Representative AP trains in response to steps of 5-pA depolarizing current lasting 500 ms from –60 mV in iNeuron recordings. (**C**) Quantitative analysis depicting the number of elicited APs in the current-clamp configuration in the 3 genotypes (WBS: 4 lines, *n* = 29 neurons; CTL: 3 lines, *n* = 40; 7Dup: 4 lines, *n* = 16) in response to increasing current steps (CTL vs. WBS: current step 35*, 45–60*, 75–85*; WBS vs. 7Dup: 35–40*, 45–50**, 55–80***, 85–95*). (**D**) Bar graph depicting the amplitude of elicited APs. (**E**–**G**) Membrane resistance was calculated in the current-clamp mode without current injection. Input resistance was calculated in voltage-clamp mode using a pulse test of 10 mV. Rheobase was calculated as the minimum current required to elicit 1 AP. (**H**) Bright-field images of isogenic iNeurons. Scale bars: 200 μm. (**I**) Representative AP trains in response to steps of 5-pA depolarizing current lasting 500 ms from –60 mV in isogenic iNeurons. (**J**) Quantitative analysis depicting the number of elicited APs in the current-clamp configuration in the isogenic iNeurons (isoWBS, *n* = 23 neurons; isoCTL, *n* = 22 neurons; 7Dup, *n* = 25 neurons) (isoCTL vs. isoWBS: 15–95****; isoCTL vs. 7Dup: 25–75****, 80–95**; isoWBS vs. 7Dup: 15–95****). (**K**) Bar graph depicting the amplitude-elicited APs. (**L**–**N**) Passive properties and rheobase of the iNeurons recordings from isogenic lines, calculated as above. Data are shown as mean ± SEM and are the average of 3 independent experiments. For comparing AP frequency, we used 2-way ANOVA followed by Tukey’s multiple-comparison test, while for comparing passive properties we used 1-way ANOVA followed by Tukey’s multiple-comparison test. *P < 0.05; ***P < 0.001.

Next, we set out to assess whether the symmetrically opposite changes in neuronal differentiation kinetics are reflected in functional differences. To this end, we examined the intrinsic excitability of neurons, a key determinant of neuronal function. We performed whole-cell current-clamp recordings to quantify the number of action potentials (APs) elicited by a series of incremental steps of current injection in iNeurons plated at low density (Figure 6A). iNeurons from WBS and 7Dup patients elicited, respectively, a consistently higher and lower number of APs, compared with iNeurons from CTL, across all current steps above 35 pA (Figure 6, B and C, and Supplemental Figure 7I), in agreement with the progressive decrease in intrinsic excitability with neuronal maturation (34). In line with intrinsic excitability differences, WBS iNeurons exhibited a higher AP amplitude and a lower rheobase compared with those from 7Dup (*P* < 0.05; Figure 6, D–G). Conversely, passive properties (input resistance and resting potential) were comparable between genotypes, confirming the healthy state of the recorded neurons. To gain further insight into the altered intrinsic excitability of iNeurons, Na^+^ and K^+^ currents were recorded in the 3 genotypes (Supplemental Figure 6, A–D) and no differences were found. These data exclude a crucial contribution of the voltage-gated sodium and potassium channels in the altered excitability observed in WBS and 7Dup neurons. To confirm that the observed effect in intrinsic excitability was the result of 7q11.23 gene dosage differences rather than a mere reflection of cell line variability, we repeated the experiments with isogenic iNeurons (Figure 6H) and confirmed that isoWBS iNeurons generated a higher number of APs compared with iNeurons from either isoCTL or 7Dup across all current steps above 15 pA (Figure 6, I and J). Similarly, AP amplitude was also consistently higher in iNeurons from isoWBS compared with those from isoCTL and 7Dup, while, again, passive properties were unaltered (Figure 6, K–N). These results uncover a 7q11.23 CNV–dependent selective impact on neuronal excitability that is highly robust across patient-derived and isogenic settings.

### The REST regulon mediates dosage-dependent pathophysiological phenotypes in the isogenic line.

**Figure 7:**
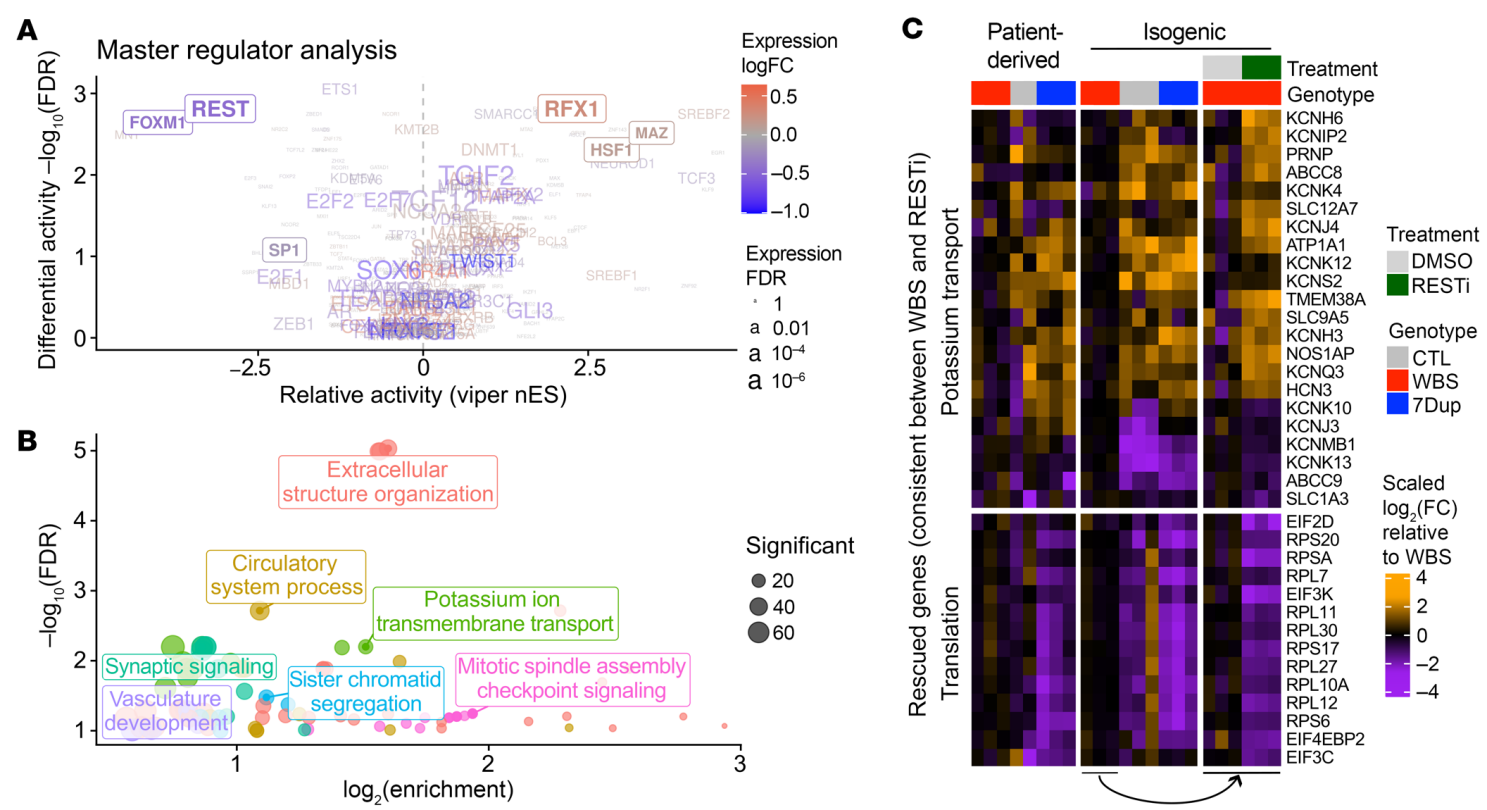
REST mediates WBS pathophysiological phenotypes. (**A**) Master regulator analysis of the 7q11.23 dosage–dependent genes based on transcription factor–curated targets. The *x* and *y* axes respectively indicate the magnitude and significance of the inferred changes in the activity, while the color and size respectively indicate the magnitude and significance of the change in expression of the factor at the RNA level. Factors in a box are consistent and statistically significant in both activity and expression. nES, normalized enrichment score. (**B**) The genes altered in WBS versus control and rescued by REST inhibition in isoWBS iNeurons are especially associated with potassium ion transmembrane transport and extracellular structural organization. (**C**) Heatmap showing potassium transport and translation-related genes that were consistently differentially expressed in WBS iNeurons and were rescued by REST inhibitor (RESTi) in isogenic lines. Note that for ease of comparison between the inhibition experiment and 7q11.23 CNV, the fold changes are shown relative to WBS.

The aforementioned CNV-dependent and symmetrically opposite endophenotypes prompted us to search for the mediating factors. We thus performed a master regulator analysis, estimating TFs’ activities based on their curated targets (see Methods). This predicted several TFs as changing in activity linearly with 7q11.23 copy number. Interestingly, several of them were also differentially expressed at the transcriptional level in a 7q11.23 copy number–dependent manner, pointing to an extensive transcriptional rewiring determined by 7q11.23 dosage (Figure 7A, TFs in boxes). Among them, we prioritized REST for functional interrogation, given its well-established role as a key regulator of neuronal differentiation by the temporal control of the expression of neuron-specific genes, including those for intrinsic excitability (26, 35). Despite its transcriptional upregulation in WBS and downregulation in 7Dup iNeurons (Supplemental Figure 6E), we found no change at the REST protein level between genotypes (Supplemental Figure 6F). Given its ranking in the master regulator analysis, we thus hypothesized that changes in the composition of REST-containing transcriptional complexes could be responsible for the transcriptional rewiring we observed. The analysis of the expression of the REST interactome (36) uncovered that nearly all reported REST interactors were transcriptionally dysregulated in a 7q11.23 dosage–dependent manner (Supplemental Figure 6G), including HDAC2, which we also previously showed to interact with GTF2I (4). To functionally validate the involvement of REST, we treated isoWBS iNeurons, which show downregulation of ion channels and other REST targets, with the REST inhibitor X5050 (37). As expected, REST inhibition rescued a set of potassium channels, including those that changed with 7q11.23 copy number (Figure 7, B and C). Interestingly, the treatment also triggered the downregulation of several important translation initiation factors and ribosomal protein transcripts that we found downregulated in 7Dup, thereby pointing to a role of REST also in the time-dependent regulation of ribosomal proteins and translational apparatus during neurogenesis (Figure 7C). Finally, we found that administration of X5050 rescued isoWBS iNeurons’ intrinsic excitability, restoring a physiological firing rate and AP amplitude comparable to those of control iNeurons (Supplemental Figure 7, A–E), while it had no impact on iNeurons from isoCTL (Supplemental Figure 7F). Next, we assessed the impact of REST inhibition on 4 WBS patient–derived lines, but failed to reproduce the effect of REST inhibition we observed in isoWBS (Supplemental Figure 7H). Expectedly, the REST inhibitor treatment of 4 CTL had no significant impact on the intrinsic excitability (Supplemental Figure 7G). However, despite not being able to reproduce the REST inhibitor effect in patient-derived lines, we could replicate the higher AP frequency in WBS compared with CTL also in this differentiation round (Supplemental Figure 7I).

## Discussion

While patient-specific approaches are a cornerstone of precision disease modeling since they afford the unique opportunity to match the specificity of clinical histories to that of molecular phenotypes, such case-control designs greatly benefit from complementary, isogenic approaches that offer a more direct route to establish causality between genetic lesions and endophenotypes. Reprogramming-based disease-modeling designs (1) that compare cell lines derived from patients and healthy individuals suffer from lower sensitivity and are inherently prone to the confounding effects of spurious, individual-specific endophenotypes arising from differences in individual genetic backgrounds and/or iPSC lines’ differentiation kinetics, rather than generalizable pathogenic mechanisms (38). In this study, we integrated patient-derived with isogenic neuronal models engineered to harbor the entire 7q11.23 CNV. This approach enabled the identification of robust molecular, cellular, and electrophysiological endophenotypes caused by 7q11.23 genetic dosage imbalances, including symmetrically opposite dynamics of neuronal differentiation, transcriptional changes, and differences in intrinsic excitability of iNeurons, as well as complex and CNV-specific patterns of posttranscriptional dysregulation.

We identified largely symmetrically opposite transcriptional changes in iNeurons, only part of which are buffered at the level of the proteome by remodeling of translation regulation. The transcriptional changes that were not buffered were enriched for genes related to neuronal transmission, in particular synaptic genes and ion channels, which is consistent with the symmetrically opposite pattern of intrinsic excitability that we observed. Master regulator analysis revealed several TFs as key targets of 7q11.23 dosage, among which we focused on the REST regulon. Its inhibition rescued the electrophysiological and underlying transcriptional changes in isoWBS, consistent with the crucial role that REST plays in regulating neuron-specific genes coding for ion channels (26, 35), which in turn determine the electrical properties of neurons and drive intrinsic excitability. The fact that the same treatment proved ineffective in patient-derived lines opens several non–mutually exclusive possibilities that further underscore the benefit of integrating both isogenic and patient-derived disease-modeling paradigms. Specifically, REST is a master regulator of neuronal differentiation and maturation whose expression and activity are temporally regulated to control orderly gene expression during neuronal differentiation (26, 35). It is thus plausible that subtle changes in the kinetics of exit from pluripotency and differentiation kinetics of individual-specific lines could have a sizable effect on REST expression and activity and hence on the sensitivity of the derived neuronal lineages to its inhibition. This is consistent with our observation of the different effects of REST inhibition on the intrinsic excitability of the individual WBS patient–derived lines tested (Supplemental Figure 7, J–M). Thus, we cannot distinguish whether the rescue with the REST inhibitor in patient-derived WBS lines failed because it intervened at not wholly aligned time points along the differentiation trajectories, as a result of different differentiation kinetics, or whether individual genetic backgrounds differentially affected the REST regulon for the endpoints we assessed.

Dysregulated ribosome biogenesis and functioning emerged recently as a common mechanism for neurodevelopmental and neurodegenerative disorders (11, 39), highlighting the importance of proper ribosome functioning for neuronal physiology. Under physiological conditions, the expression of ribosomal protein genes decreases during neuronal differentiation (27). Here, we observed a decrease in ribosomal protein transcripts in 7Dup and an increase in WBS compared with CTL, in line with symmetrically opposite kinetics of differentiation between WBS and 7Dup. Surprisingly, these differences were buffered at the level of translation. The coordinated translation of ribosomal protein mRNAs and other translation-related genes with the 5′ TOP motif is regulated by the mTOR pathway (30) and it was shown that mTOR stimulation partially rescued NDD endophenotypes characterized by dysregulation of ribosomal biogenesis (reviewed in ref. 11). We thus assessed the activity of the mTOR pathway and, concordantly with the symmetrically opposite translational regulation of TOP mRNAs, found higher activity of the mTOR in 7Dup compared with isoCTL and isoWBS, when profiling phosphorylation of RPS6 as the readout. The higher mTOR activity in 7Dup is also in line with the overactivation of the mTORC1 in ASD (40), whose prevalence in 7Dup patients is at least 20% (2, 41). However, we found the opposite trend for 4EBP, where both the total and phosphorylated forms were inversely affected by 7q11.23 dosage. The uncoupling of p-4EBP and p-RPS6 has already been documented in neurogenesis. For example, while p-4EBP marks neural stem cells undergoing mitosis and NPCs at the ventricular surface, p-RPS6 marks more differentiated cells migrating away from the ventricle (9, 42, 43). Interestingly, it was shown that NPCs marked with p-4EBP had higher levels of ribosomal protein mRNAs compared with more mature neurons marked with p-RPS6, but the difference was buffered at the level of translation in cortical brain organoids (43). Thus, higher p-RPS6 in 7Dup compared with isoWBS, and the opposite trend for 4EBP — a higher expression of 4EBP and higher p-4EBP in isoWBS than 7Dup — may reflect merely the difference in the kinetics of differentiation between the two.

An alternative hypothesis is that genotype-specific differences in mTOR activity could also be directly traced to the genes from the WBSCR, rather than being byproducts of a primary effect on differentiation kinetics. Thus, WBSCR22, an 18S rRNA methyltransferase involved in pre-rRNA processing and ribosome 40S subunit biogenesis (44), could affect ribosomal biogenesis per se, which in turn would lead to dysregulated mTOR activity as it was shown that ribosomal protein knockdowns reduce the basal activity of the mTOR pathway, but do not prevent its BDNF-mediated activation (assessed by the p-S6 levels in ref. 45). Likewise, the WBSCR-encoded protein DNAJC30 interacts with ATP synthase, alters mitochondrial functioning in neurons (17), and alters ATP levels, which could directly affect the mTOR activity. The most plausible scenario is that both hypotheses are correct and that the activity of the mTOR pathway is both under direct and indirect (i.e., mediated via changes in differentiation kinetics) effects of WBSC dosage, in the inherently intertwined impact of such a dosage-sensitive array of genes.

Finally, regarding buffering, an interesting hypothesis is that the molecular mechanism underlying buffering in 7Dup and WBS are distinct, which would explain the differences in both the extent and the pool of buffered mRNAs. While the mTOR pathway, probably through LARP1 (31), is responsible for the buffering in 7Dup in agreement with the hyperactivation of the mTOR pathway observed in 7Dup and ASD models, buffering in isoWBS could be a mere consequence of asymmetric kinetics of differentiation, as a decreased translation of TOP mRNAs was observed in the less mature cells during neurogenesis.

The complexity of ribosomal biogenesis and mTOR regulation in 7q11.23 CNVs underscores the importance of defining the scale at which it makes most sense to probe CNVs mechanistically, by altering single genes or the CNV as a whole. We have tackled this challenge here through the integration of 3 regulatory layers across 2 modeling designs, focusing on the CNV as a whole.

Interestingly, REST inhibition in isoWBS rescued the expression of key translation genes, including some ribosomal proteins, suggesting that the transcriptional and translational aspects are heavily intertwined. Intrinsic excitability decreases during neuronal differentiation, which is regulated by the REST regulon. REST expression decreases during neuronal differentiation in development (26, 35) along with the decreased expression of ribosomal proteins (27), while synaptic and ion channel genes instead increase (26). It is therefore attractive to relate all the changes we describe to the delay and acceleration in differentiation, respectively observed in WBS and 7Dup, both in the present and in our recent work focused on the role of *GTF2I* dosage (5). Indeed, an increasing body of evidence from us and others suggests that changes in the dynamics of neuronal differentiation (acceleration and delay) are a major point of convergence across NDDs, despite differences in the underlying molecular mechanisms (46–48).

In conclusion, we show that systematic investigation of multiple molecular layers at omics resolution (transcriptome, translatome, proteome) integrated with functional endophenotypes (differentiation dynamics and excitability) can address an outstanding question of the disease modeling field, namely, how phenotyping at the level of the transcriptome (arguably the most proximal and tractable layer) reverberates through more distal endophenotypes in pathophysiologically meaningful disease-relevant cell types. Thus, starting from the pair of 7q11.23 CNVs featuring a paradigmatic suite of symmetrically opposite and shared manifestations, we uncover several dosage-dependent endophenotypes that are well-established proxies of cognitive-behavioral phenotypes, revealing a multilayered interplay between kinetics of differentiation and neuronal function with transcriptional and translational control that can productively inform the study of other NDDs.

## Methods

### Sex as a biological variable.

Sex was not considered as a biological variable.

### Human samples.

In this study, we used patient-derived iPSC lines that we have previously generated and reported (4, 5, 19). Briefly, we used the following lines: DUP01 (CF; male [M]), provided by P. Prontera (University of Perugia); DUP02 (242; female [F]), DUP03 (809; M), CTL01 (339-1; F), CTL04 (809-1; F), WBS01 (339; M), WBS02 (316; F), WBS03 (361; M), and WBS04 (306; F), all provided by G. Merla (Telethon Biobank); DUP04 (103, M) provided by F. Kooy (University of Antwerp); CTL02 (MIFF; M) provided by P. Andrews (University of Sheffield); and CTL03 (Bu1Cre; M) provided by G. Mostoslavsky (Boston University). Isogenic lines were generated from the DUP02 line. Patch-clamp recordings were performed on the following cell lines: CTL01, CTL02, CTL03, CTL04, WBS01, WBS02, WBS03, WBS04, DUP01, DUP02, DUP03, and DUP04. Transcriptomics and proteomics were performed on DUP01, DUP02, DUP03, CTL01, CTL02, WBS01, WBS02, and WBS04; in addition, for transcriptomics we also used CTL03, while for proteomics we used CTL04. Ribosomal profiling and immunostainings on organoids were done only on isogenic lines.

### Generation of isoCTL and isoWBS.

The isogenic lines, isoCTL and isoWBS, were generated in 2 consecutive rounds of CRISPR/Cas9 genome editing by cotransfecting purified Cas9 protein and specific gRNAs. For the generation of isoCTL, we exploited the duplication of WBSCR in a 7Dup patient (242K), introducing only 1 gRNA that cut both duplicated WBSCRs, and we screened for the combination where the 5′ end of the first WBSCR fused to the 3′ end of the second WBSCR, thus generating 1 complete WBSCR on the place of initial duplication (Figure 1A). For the generation of the isoWBS instead, we used 2 gRNAs, centromeric and telomeric, which delineate the whole WBSCR. We designed forward primers for gRNAs containing universal forward primer for T7 promoter (in blue), thus allowing in vitro transcription. In vitro transcription was performed on a purified PCR template for gRNA. The primers for isoCTL were F1 and R1, while for isoWBS, we designed F_CE and R_CE for a centromeric cut and F_TE and R_TE for a telomeric cut. The gRNAs were designed by using the MIT CRISPR design tool. Primers for gRNAs were (T7 promoter sequence underlined): F1-TAATACGACTCACTATAGGAATCTCAGGTCCGCCCCA; R1-TTCTAGCTCTAAAACTGGGGCGGACCTGAGATTC; F_CE-TAATACGACTCACTATAGCGAAGTTGTTTTCCGAGGC; R_CE-TTCTAGCTCTAAAACCGCCTCGGAAAACAACTTCG; F_TE-TAATACGACTCACTATAGAGCGCCTTGATCCGATCACT; R_TE-TTCTAGCTCTAAAACAGTGATCGGATCAAGGCGCT.

PCR reactions (5 μL Phusion HF buffer 5×, 0.25 μL Phusion polymerase, 0.5 μL 10 mM dNTPs, 1 μL Tracer Fragment + T7 primer Mix, 1 μL F/R oligonucleotide mix [0.3 μM] and 17.25 μL H_2_O) were done as follows: initial denaturation of 98°C for 10 seconds; 32 cycles of denaturation at 98°C for 5 seconds and annealing at 55°C for 15 seconds; followed by final extension at 72°C for 60 seconds. In vitro transcription (8 μL of NTP mix [100 mM ATP, GTP, CTP, UTP], 6 μL PCR template of gRNA, 4 μL TranscriptAid Reaction Buffer 5×, 2 volumes TranscriptAid Enzyme Mix) was performed for 2 hours at 37°C, followed by DNase treatment (1 μL of DNase I, 1 U/μL) for 15 minutes at 37°C. The product was visualized on agarose gel and purified with 5 M ammonium acetate. Briefly, one-half volume of 5 M ammonium acetate and 3 volumes of 100% ethanol were added to the samples, which were precipitated for 1 hour at –80°C and then spun for 30 minutes at 20,000*g* and 4°C. The RNA pellets were dried for 25 minutes and resuspended in 200 μL RNase-free H_2_O.

We cotransfected 4 × 10^5^ cells (seeded per well of a 6-well plate) with 20 μg Cas9 and 10 μg gRNA. Cas9 was resuspended in Cas9 transduction buffer 5× (500 mM NaCl, 25 mM NaH_2_PO_4_, 250 mM NDSB-201, 150 mM glycerol, 75 mM glycine, 1.25 mM MgCl_2_, and 1 mM 2-mercaptoethanol, pH 8.0) to obtain a final concentration of 3.88 μg/μL. The Cas9/gRNA mix was incubated at 37°C for a maximum of 10 minutes and then electroporated. The electroporation was performed using the Neon Transfection System (MPK10096, Thermo Fisher Scientific). The iPSCs were pretreated for 2 hours with 5 μM RI, detached with Accutase (GIBCO/Thermo Fisher Scientific) to obtain single-cell suspension, and resuspended in buffer R supplemented with Cas9/gRNA mix for a total volume of 120 μL for 4 × 10^5^ cells. The electroporation was performed at 1300 volts for 20 ms and 2 pulses. The cells were seeded in mTeSR supplemented with RI. Electroporated cells were single-cell sorted in 96-well plates by using DAPI, 24 hours after electroporation. The medium was not changed for the first 5 days, and from the sixth day on, only half of the medium was changed every second day. We started to change the whole medium only when the colonies started to be visible. When confluent, cells were split first into 48-well plates, then 12-well plates, and screened when they reached confluence in 6-well plates. We obtained 17 clones for isoCTL and 8 for isoWBS, which were further analyzed by digital PCR and FISH for WBSCR CNV.

### PiggyBac transposon system.

For robust and rapid glutamatergic neuron differentiation, we used the PiggyBac transposon system for *Ngn2* delivery to the cells, as previously described (19). Briefly, mouse *Ngn2* cDNA, under tetracycline-inducible promoter (tetO), was transfected into iPSCs by an enhanced PiggyBac (ePB) transposon system (20). For each iPSC line, 4 × 10^5^ cells were electroporated with 2.25 μg of the ePB construct carrying the inducible *Ngn2* cassette and 250 ng of the plasmid encoding transposase for the genomic integration of the inducible cassette. Electroporation was performed using the Neon Transfection System. iPSCs were selected using 5 μg/mL blasticidin (R21001, Gibco) for 5 days, and stable iPSC lines were stocked.

### Ngn2-driven neuronal differentiation.

To obtain cortical glutamatergic neurons (iNeurons), iPSCs were dissociated with Accutase and plated on Matrigel-coated plates (final 2% v/v, Corning) in mTeSR or TeSR-E8 (STEMCELL Technologies) supplemented with 5 μM Y-27632 (Sigma-Aldrich). iPSCs were then cultured in MEM1 (DMEM/F12 1:1; Euroclone/Gibco) supplemented with 1% nonessential amino acids, 1% N2, 10 ng/mL BDNF (Peprotech), 10 ng/mL NT-3 (Peprotech), 0.2 μg/mL laminin (Roche), 2 μg/mL doxycycline hydrochloride, 100 U/mL penicillin, and 100 μg/mL streptomycin) for 2 days. On the second day of MEM1, cells were selected with 1 μg/mL puromycin to ensure that only the cells with an *Ngn2*-inducible cassette will survive. For patient-derived iNeurons, after 2 days of MEM1, the medium was changed to Neurobasal medium (NB, Thermo Fisher Scientific) supplemented with 10 ng/mL BDNF, 10 ng/mL NT-3, B27 (1:50), GlutaMax (Gibco, 1:100), 100 U/mL penicillin, and 100 μg/mL streptomycin, which was previously conditioned on mouse astrocytes for 24 hours. Half of the media was changed every other day. Instead, for isogenic iNeurons after 2 days of MEM1, media were changed to Neurobasal Plus medium (NB-Plus) composed of B-27 Plus Neuronal Culture System (Thermo Fisher Scientific) supplemented with 0.25% GlutaMax (Thermo Fisher Scientific), 2 μg/mL doxycycline hydrochloride, 100 U/mL penicillin, and 100 μg/mL streptomycin. The media were changed twice a week. On days 7–8, cells that already acquired a neuron-like shape were dissociated with Accutase, counted, and seeded into plates coated with 15 μg/mL of poly-D-lysine at a density of 1 × 10^6^ cells/well of a 6-well plate, 2 × 10^6^ in 6-cm dishes, or 4 × 10^6^ in 10-cm dishes (Nunc Edge plates, Thermo Fisher Scientific) in conditioned NB or NB-Plus. Half of the media was changed twice a week until days 30–35. iNeurons were grown in 3% oxygen.

For electrophysiological recordings (intrinsic excitability), iPSC differentiation was performed in a normal incubator environment (20% oxygen, 5% carbon dioxide) in the presence of mouse astrocytes (1:1) from day 7 onwards, on day 2 media was changed to Neurobasal A and DMEM/F12 1:1 (STEMCELL Technologies) supplemented with 0.5% N2, 1% nonessential amino acids, 10 ng/mL BDNF, 10 ng/mL NT-3, 0.2 μg/mL laminin, 1% Culture 1 (Gibco), 2.5% FBS (Sigma-Aldrich), and 2 μg/mL doxycycline hydrochloride.

For spontaneous excitatory postsynaptic current recording instead, we added 1:1 rat astrocytes on day 2, and medium from days 3 to 7 in NB-Plus supplemented with B-27, 10 μg/mL GlutaMax, 0.1 μg/mL primocin (InvivoGen), 10 ng/mL BDNF, 10 ng/ml NT-3, and 2 μg/mL doxycycline hydrochloride. From day 10 onwards, the medium was supplemented with 2.5% FBS.

### OMICs analysis.

The code underlying the omics analyses and related figures, as well as re-usable data objects, are available at https://github.com/plger/7q11ngn2 Unless specified otherwise, the expression heatmaps show log_2_FC with respect to the controls of each data set, with the color scale based on the central 98th percentiles to avoid distortion by outliers, as implemented in the sechm package. All data are available at 7q11.23 Explorer (https://ethz-ins.org/7q/), a web server that allows browsing the data.

### RNA-seq analysis.

Quantification was done using Salmon 0.9.1 (49) on the Ensembl 92 transcriptome. Only protein-coding transcripts were retained, and counts were aggregated at the level of gene symbols. A line from a WBS patient with an atypical deletion was profiled alongside other patient-derived lines (and is deposited), and was included for normalization and dispersion estimates, but was excluded from differential expression and downstream analysis, and not presented in this paper for the sake of simplicity. Only genes with at least 20 reads in at least several samples corresponding to 75% of the smallest experimental group were included in the analysis. Differential expression analysis was done using DESeq2 (50), and for pairwise comparisons between groups, fold changes were shrunk using the apeglm method (51). In addition, a regression on 7q11.23 copy numbers was performed. Unless the analysis is specified, DEGs include the union of genes significant across these analyses.

For the merged analysis of isogenic and patient-derived data sets, we first performed surrogate variable analysis to account for technical vectors of variation using the sva package (52) on variance-stabilized data (as implemented in the SEtools package and benchmarked in ref. 53), and included the 2 variables in the differential expression model. Unless specified otherwise, genes were considered differentially expressed in the merged analysis if they showed at least a 30% difference and had an FDR of less than 0.01 (in any of the comparisons), and were additionally differentially expressed in both data sets with an FDR of less than 0.5.

### Proteomics analysis.

Analyses were performed at the level of protein groups, using the median of the top 3 peptides. The intensity signals across technical replicates were averaged. Potential contaminants and protein groups with more than 4 missing values per data set were excluded. Variance-stabilizing normalization and imputation using the minProb method were used, as implemented in the DEP package (54). Surrogate-variable analysis was performed before running differential expression analysis via limma/eBayes (55), again using pairwise comparisons between groups or a regression on copy numbers.

### Ribosome footprinting analysis.

Trimmed reads were mapped with STAR v2.5.2b (56) using the Ensembl 92 transcriptome as a splice junction guide. One sample (7Dup) was excluded due to poor mapping rate and codon periodicity. Reads mapping to coding sequences were quantified at the gene level using featureCounts v1.5.1 (57). Differential expression analysis was performed as described for RNA-seq. In addition, differential TE was assessed by fitting a ~Replicate+Genotype+SeqType+SeqType:Genotype model using DESeq2 and testing for the interaction terms.

### Integrative analysis across layers.

For the integrative analysis of translation, we first restricted ourselves to genes that had an FDR of less than 0.05 and a |logFC| of greater than 0.25 at the transcriptome or proteome or had aggregated significance (Fisher’s method) across the 3 layers. We then took genes that were significant and in the same direction in both the transcriptome and the proteome, in order to establish the normal relationship between RNA and protein logFC (fitting a robust linear model without intercept) for forwarded genes. The genes were fairly well distributed along the diagonal (*r* = 0.91), and their spread was used to establish a 2-SD interval in which genes were classified as forwarded. For other genes, the residuals were considered as evidence of gene-specific posttranscriptional changes. We next repeated this procedure fitting the residuals on the TE logFC for genes with a TE *P* value of less than 0.01, and again observed a fair correlation (*r* = 0.8). Other genes within the 95% confidence interval around the diagonal were then considered regulated at the level of translation, as their TE pattern explained the RNA-protein discrepancies.

For the clustering across layers, for each layer and condition we first scaled the genes’ logFCs by unit variance (without centering), and assigned a value of 1 if the median was above 0.2 and all individual logFCs were in the same direction, a value of 0.5 if the median was above 0.2 but individual scores were not in the same direction, and a value of 0 if below threshold (the same for downregulated genes). We then concatenated the RNA and protein scores and performed *k*-means clustering with 8 centers. Similar clusters were then grouped manually.

### Enrichment analysis.

Gene Ontology (GO) overrepresentation analysis of genes found differentially expressed via sequencing was performed using the goseq package (58) to account for length biases, using the genes passing the aforementioned filtering (and with some GO annotation) as background and restricting to terms of annotated to at least 10 and not over 1000 genes. To minimize redundancy in visual representations, significant terms were clustered using *k* means (using the elbow of the variance explained plot to choose *k*) on the binary matrix of gene-term membership. Individual terms were then colored by cluster, and the most significant term of each cluster was used to label the clusters.

### Master regulator analysis.

The master regulator analysis was based on the DoRothEA v0.0.25 regulons, assigning interaction likelihoods weighted according to the approximative AUC of the different interaction categories in their benchmark (59). Per-sample TF activity was then estimated using the viper package (60) based on the sva-corrected data (if applicable), and differential activity assessed using limma, blocking for data set effects. In addition, a differential TF activity analysis was performed on the RNA-seq differential expression statistic using msviper, and only TFs that were consistent between the 2 analyses were considered.

The rest of the methods can be found in the supplemental material.

### Statistics.

All statistical methods used are indicated in the appropriate figure legends. In brief, 1-way ANOVA followed by Tukey’s multiple-comparison test was used for the statistical analysis of the results presented in Figures 1E, 2B, 2D, and 5I and Supplemental Figures 1C and 4I, and for comparing passive properties in Figure 6, D–G and K–N and Supplemental Figure 7, B–E. Two-way ANOVA followed by Tukey’s multiple-comparison test was used for the graphs in Figure 5, B–G and Supplemental Figure 4, A–F, and for comparing AP frequency in Figure 6, C and J and Supplemental Figure 7, A and F–I. The significance level was set at *P* less than 0.05: *P < 0.05; **P < 0.01; ***P < 0.001. GraphPad Prism 10 was used for all statistical analyses. Statistics applied for the OMICS analyses (transcriptomics, proteomics, ribosomal profiling) is described in corresponding Methods sections.

### Study approval.

Participation of the patients and healthy control individuals along with skin biopsy donations and informed consent procedures were approved by the ethics committees of the hospitals where the samples were collected. Relevant ethics approvals are referred to in the original publications reporting their first use and/or use of the derivations (4, 5, 19).

### Data availability.

The transcriptomics data were deposited in the NCBI Gene Expression Omnibus (GEO) under accession number GSE261692. The mass spectrometry proteomics data have been deposited in the ProteomeXchange Consortium via the PRIDE partner repository with accession number PXD035276 for isogenic and PXD038156 for patient-derived lines. All data can additionally be browsed on our database at https://ethz-ins.org/7q/ The list of amplified genes at Chr14 is provided in Supplemental Table 1. Transcriptomics results obtained in patient-derived and isogenic lines are shown in Supplemental Table 2. The genes included in clusters across the 3 regulatory layers are listed in Supplemental Table 3. Values for all data points in graphs are reported in the Supporting Data Values file.

### Code availability.

The code is available at the following link: https://github.com/plger/7q11ngn2

## Author contributions

M Mihailovich, PLG, RS, and GT designed the experiments. M Mihailovich, RS, PLG, ET, GDA, AVF, ST, SF, AN, FT, AS, IB, AV, DC, YL, RA, RN, TB, MTR, NC, DA, SR, UC, NNK, DP, and M Matteoli performed the experiments and analyzed the data. M Mihailovich, PLG, RS, and GT wrote the manuscript. All authors contributed to the article and approved the submitted version. The order of the co–first authors was determined based on their efforts and contributions to the study.

## Supplementary Material

Supplemental data

Unedited blot and gel images

Supplemental table 1

Supplemental table 2

Supplemental table 3

Supporting data values

## Version 1 07/15/2024

Electronic publication
