# Supplementary material for: Multiscale modeling uncovers 7q11.23 copy number variation–dependent changes in ribosomal biogenesis and neuronal maturation and excitability
Source: J Clin Invest. 2024 Jul 15;134(14):e168982. doi: 10.1172/JCI168982 (PMC11245157; doi:10.1172/JCI168982)
Supplement: Supplemental data [file jci-134-168982-s088.pdf]

Supplemental material

Multi-scale modeling uncovers 7q11.23 copy number variation-dependent alterations in ribosomal biogenesis, neuronal maturation and excitability

Marija Mihailovich[1,2,3§*], Pierre-Luc Germain[1,4,5*], Reinald Shyti[1,2*], Davide Pozzi[6,7], Roberta Noberini[1], Yansheng Liu[,8,9], Davide Aprile[2,10], Erika Tenderini[1,11] Flavia Troglio[1,2,10], Sebastiano Trattaro[1,2,10], Sonia Fabris[12], Ummi Ciptasari[13], Marco Tullio Rigoli [1,2,10], Nicolò Caporale[1,2,10], Giuseppe D'Agostino[1,14], Filippo Mirabella[2], Alessandro Vitriolo[1,2,10], Daniele Capocefalo[2,10], Adrianos Skaros[1,2], Agnese Franchini[1], Sara Ricciardi[15,16], Ida Biunno[17], Antonino Neri[10,12], Nael Nadif Kasri[13], Tiziana Bonaldi[1,10], Rudolf Aebersold[8], Michela Matteoli[6,7] and Giuseppe Testa[1,2,10§#]

[1]IEO, European Institute of Oncology IRCCS, Milan, Italy

[2]Human Technopole, Milan, Italy

[6]Department of Biomedical Sciences, Humanitas University, Milan, Italy

[7]IRCCS Humanitas Research Hospital, Milan, Italy

[8]Department of Biology, Institute of Molecular Systems Biology, Federal Institute of Technology (ETH) Zurich, Zurich, Switzerland

[10]Department of Oncology and Hemato-Oncology, University of Milan, Milan, Italy.

[12]Hematology Unit, Fondazione IRCCS Ca' Granda Ospedale Maggiore Policlinico, Milan, Italy.

[13]Department of Cognitive Neurosciences, RadboudUmc, Donders Institute for Brain Cognition and Behaviour, Nijmegen, the Netherlands

[15]Department of Biosciences, University of Milan, Milan, Italy.

[16]National Institute of Molecular Genetics, Fondazione Romeo ed Enrica Invernizzi, Milan, Italy

[17]Integrated Systems Engineering Srl, c/o OpenZone, Bresso, Milan, Italy

[*]These authors contributed equally

[§]Senior authors

[#]Correspondence: Giuseppe Testa, Head of Neurogenomics Research Centre; Testa Group - Neurogenomics Research Centre; Human Technopole, Palazzo Italia, Viale Rita Levi-Montalcini, 1; 20157 Milan, Italy; Mobile: +39 0230247166; email: giuseppe.testa@fht.org

Present addresses:

[3]Institute of Molecular Genetics and Genetic Engineering (IMGGE)-University of Belgrade, Belgrade, Serbia

[4]Computational Neurogenomics, D-HEST Institute for Neuroscience, ETH Zürich, Switzerland

[5]DMLS Lab of Statistical Bioinformatics, University of Zürich, Switzerland

[9]Department of Pharmacology, Yale University School of Medicine, New Haven, CT, USA

[11]Telethon Institute of Genetics and Medicine (TIGEM), Pozzuoli, Naples, Italy,

[14]Plasticell Limited, Stevenage Bioscience Catalyst, Gunnels Wood Road, Stevenage SG1 2FX, United Kingdom

Conflict-of-interest statement: The authors have declared that no conflict of interest exists.

Supplemental Information

Genetic characterization of the isogenic lines

To verify the genetic background of our lines, we carried out Whole Exome Sequencing (WES) on the isogenic lines and a whole genome sequencing (WGS) on the 'mother' 7Dup line we used to generate the isogenic lines, albeit on the higher passage since we did not have any cells of the passage from which the isogenic lines were generated. We performed the canonical variant calling using the Sarek workflow (https://nf-co.re/sarek/3.2.3). Variants were called with DeepVariant (https://www.nature.com/articles/nbt.4235) and annotated using the Variant Effect Predictor (VEP) on all the coding exons at our disposal. Since the WES and WGS do not completely overlap, we selected variants falling in curated coding exons in refSeq, that were selected by downloading coding exons intervals from the UCSC Genome Browser on June 2023. We focused our attention on all genes that were associated with ASD in the SFARI Database (the complete list of genes is available in the SFARI_Autism_Genes.tsv, downloaded on March 2023, and it contains 1128 genes) and retained variants using the following quality check: Filter - PASS according to DeepVariant; Genotype Quality (GQ) - 20 or higher and Coverage (DP) - 10 or higher. We found in a total of 876 variants for the original 7Dup line, 151 variants for isoCTL, 170 variants for isoWBS, clone #1, and 167 variants for isoWBS, clone #15 (which was used in our study). With only one exception described below, we found no variants whose impact is reported as 'HIGH IMPACT' in the VEP guidelines for interpreting variants: https://www.ensembl.org/info/genome/variation/prediction/predicted_data.html. Instead, all variants with a 'MODERATE' impact show contradictory results, either because they exhibit a high frequency in different population databases (GNOMAD, ExAc, 1000Genomes) or because they are reported to be 'benign' or 'likely benign' according to ClinVar. Moreover, they show conflicting results for their prediction of pathogenicity

according to different predictors (SIFT, PolyPhen). We, hence, didn't consider these mutations as potentially synergistic with 7q11.23 CNV in driving the observed phenotype.

The only exception we found is a homozygous start-lost vitamin-D receptor variant found in the original 7Dup line, with dbsnIP rsID: rs2228570 (https://www.ncbi.nlm.nih.gov/snp/?term=rs2228570), with no clear relation to the described phenotypes. Moreover, since this variant appears only in the 'mother' line and not in the isogenic lines, we speculate that the mutation occurred at a later passage concerning when the isogenic lines were generated. We also inspected for possible interactions between proteins encoded by genes located at Chr14 and those within the WBSCR (Supplemental Figure S2E-F). We then reconstructed the protein-protein interaction network between the WBSCR and Chr14 genes. We obtained a widely unconnected network made of 20 PPIs across 30 proteins composed of 11 WBSCR, and 19 Chr14 proteins, as shown in Supplemental Figure S2E. Furthermore, we overlaid information from differential gene expression and differential protein abundance coming from isogenic vs 'mother line', and retained only fold-changes with FDR equal to or smaller than 0.05. Out of 19 Chr14 genes interacting with WBSCR genes, 5 showed significant, yet, small variations in expression (**-0.534** <= logFC >= (1)**0.527**) and only 3 showed a differential protein abundance (-0.329 <= logFC => 0.457). These findings point to no significant impact of Chr14 trisomy on the described 7q11.23 phenotypes.

Supplemental Methods

iPSC culture

iPSC lines were cultured on plates coated with human-qualified Matrigel (BD Biosciences; dilution 1:40 in DMEM-F12) in mTeSR or TeSR-E8 medium (StemCell Technologies) supplemented with penicillin 100 U/ml and streptomycin (100 µg/ml). Cells were passaged with ReLeSR (StemCell Technologies) or Accutase (Sigma). For single-cell culture, cells - were plated in mTeSR or TeSR-E8 medium supplemented with 5 µM Y-27632 (Rock inhibitors (RI), Sigma). Cells were grown at 3% oxygen.

Fluorescence in situ hybridization (FISH) analysis

A co-hybridization using a specific clone covering the *ELN* locus at 7q11.23 and a specific alpha-satellite as a control probe for ploidy status was performed to investigate the *ELN* gene pattern iPSCs. Specifically, CTD-2510G2 BAC clone for the *ELN* gene and 7 Alpha Satellite probes were used for the identification of the gene copy number. Considering that the distance between two identical WBSCR on a duplicate chromosome is approximately the same as WBSCR length (~ 1,8 Mb), the 7Dup signal pattern was resolved in interphase nuclei (metaphase chromosomes are thousands of times more compacted than interphase chromosomes). The BAC clone was selected using the University of California Santa Cruz Genome Browser Database (http://genome.ucsc.edu/) and was tested on normal human metaphase cells to verify the absence of cross-hybridization while the alpha-satellite probes were kindly provided by Dr. M. Rocchi, University of Bari, Italy. FISH experiments were performed as previously described, with minor modifications (1).

Digital PCR

80 ng of DNA was amplified in a reaction volume of 15 µl containing the following reagents: 7.5 µl of "QuantStudio™ 3D Digital PCR Master Mix v2, Thermofisher", 0.75 µl of "TaqMan Copy Number Assays, SM 20x, Thermofisher" FAM labeled and 0.75 µl of "TaqMan Copy Number Reference Assay 20x, Thermofisher" VIC labeled (for details see below). The mix was loaded on a chip using the QuantStudio 3D Digital PCR Chip Loader. The chips were then loaded on the Proflex PCR System (Thermofisher), and the PCR was carried out using a pre-PCR step of 10 min at 96°C, followed by 39 cycles of 2 min at 60°C and 30s at 98°C, followed by 2 min at 60°C.

Data were analyzed with "QuantStudio 3D Analysis Suite Cloud Software". The entire process was performed by the qPCR-Service at Cogentech-Milan, Italy. TaqMan Copy Number Assays (FAM): *CLIP2* Hs03650293_cn, *BAZ1B* Hs04952131_cn andEIF4H Hs01632079_cn; TaqMan Copy Number Reference Assay: TERT (VIC) catalog number 4403316.

Short Tandem Repeat (STR) analysis

STR analysis was done with GenePrint 10 System (Promega) according to the manufacturer's instructions. STR loci consist of short (3-7 nt), repetitive sequence elements. They are well distributed with the human genome and represent a rich source of highly polymorphic markers that can be detected by PCR. GenePrint 10 System allows co-amplification and 3-color detection of nine human loci, providing a genetic profile with a random match probability of 1 in 2.92 x 10^9. PCR master mix contained 5x master mix, 5x primer master mix, and 2 ng of genomic DNA. The thermal cycling protocol was one recommended by the manufacturer (96°C -1 min; 30 cycles of 94°C -10 sec, 59°C -1 min, 72°C -30 sec; 60°C -10 min; 4°C).

Karyostat

The KaryoStat™ (Thermo Fisher) assay allows for digital visualization of chromosome aberrations with a resolution similar to g-banding karyotyping by relying on 150k SNP probes across the human genome. The size of structural aberration that can be detected is > 2 Mb for chromosomal gains and > 1 Mb for chromosomal losses. Genomic DNA was extracted from pellets corresponding to 2 x 10^6 cells by using the Genomic DNA Purification Kit (Catalog #: K0512) and quantified using the Qubit™ dsDNA BR Assay Kit (Catalog #: Q32850). 250 ng of total gDNA was used to prepare the GeneChip® for KaryoStat™ according to the manual, and the array was used to look for SNPs, copy number variants and single nucleotide polymorphisms across the genome.

Generation of neural progenitor cells with STEMdiff™ Neural Induction Medium

iPSCs were dissociated with Accutase (GIBCO, Thermo Fisher Scientific) and plated on Matrigel-coated plates (final 2% v/v, Corning) directly in STEMdiff™ Neural Induction Medium (#05839, Stem cell technologies) supplemented with 5 μM Y-27632 (Sigma). Cell medium was changed daily till day 5 when the neural progenitor cells were analyzed.

Cortical organoids generation

Cortical brain organoids were differentiated until day 18 or 50, following the protocol described in (2), with minor modifications to improve its efficiency as we showed previously (3). Since we optimized the protocol to avoid the use of mouse embryonic fibroblast (MEF) for stem cell culture, the following procedures were followed: when the hiPSC line reached 80% confluency in a 10 cm dish, colonies were dissociated with Accutase and centrifuged to remove the enzymatic suspension. After resuspension in TeSR/E8 medium supplemented with 5 μM ROCK inhibitor cells were counted with a TC20 automated cell counter (Biorad) and

resuspended to get a final concentration of 2 x 10^5 cells/ml. 100 µl/well of cell suspension was seeded into ultra-low attachment PrimeSurface 96-well plates (SystemBio) and then the plates were centrifuged at 850 rpm for 3 minutes to promote the formation of embryoid bodies (EB). The day of the EB generation is referred to as day -2. On day 0 neuronal induction media was added, consisting of 80% DMEM/F12 medium (1:1), 20% Knockout serum (Gibco), 1 mM non-essential amino acids (Sigma), 0.1 mM cell culture grade 2-mercaptoethanol solution (Gibco), GlutaMax (Gibco, 1:100), penicillin 100 U/ml, streptomycin (100 µg/ml), 7 µM Dorsomorphin (Sigma) and 10 µM TGFβ inhibitor SB431542 (MedChem express) for promoting the induction of neuroectoderm. From day 0 to day 4, medium change was performed every day, while on day 5 neuronal differentiation medium was added, consisting of neurobasal medium (Gibco) supplemented with B-27 supplement without vitamin A (Gibco, 1:50), GlutaMax (1:100), penicillin 100 U/ml, streptomycin (100 µg/ml), 20 ng/ml FGF2 (Thermo) and 20 ng/ml EGF (Thermo) until day 25. From day 25-43, the neuronal differentiation medium was supplemented with 20 ng/ml BDNF and NT3 (Peprotech). From day 43 onwards no growth or neuronal maturation factors were added to the medium.

Cortical organoids processing and immunostaining

Cortical organoids were harvested on day 18 or 50, fixed overnight at 4 °C in paraformaldehyde 4% PBS solution (Santa Cruz). After rinsing with PBS, organoids were embedded in 2% low melting agarose dissolved in PBS; upon agarose solidification, blocks were put in 70% ethanol and kept at 4 °C before paraffin embedding, sectioning, and routine hematoxylin/eosin staining. Deparaffinization and rehydration were achieved by consecutive passages of 5 minutes each in the following solutions: 2 x histolemon (Carlo Erba), 100% ethanol, 95% ethanol, 80% ethanol and water. Sections were then incubated for 45 min at 95°C in 10 mM Sodium citrate (Normapur)/ 0,05% Tween 20 (Sigma) buffer for simultaneous antigen retrieval and

permeabilization; then left to cool for at least 2 hours at RT. To immunolabel the markers of interest, a blocking solution made of 5% donkey serum (ImmunoResearch) in PBS was applied for 30 minutes to the slides, while primary antibodies anti-KI67 (Abcam ab15580; 1:500); anti-PAX6 (Biolegend 901301; 1:300); anti-CTIP2 (abcam ab18465; 1:500) were diluted in the blocking solution were subsequently added, performing overnight incubation at 4°C. Secondary antibodies and DAPI were diluted in PBS and applied to the sections for 1 hour and 5 minutes respectively. After each incubation step, 3 x 5 minutes of washing steps with PBS buffer were performed. After a final rinse in deionized water, slides were air-dried and mounted using a Mowiol mounting medium. The following primary antibodies and dilutions were used; KI67 (1:500 Abcam) and PAX6 (1:250 Abcam), CTIP2 (1:500 Abcam).

Cortical organoids image acquisition and processing

Image acquisition of 5 μm organoids paraffin sections was done in Leica DM6 B widefield microscope. KI67, PAX6 and CTIP2 quantification was done by counting the number of positive cells and normalized against the number of DAPI positive cells using automated cell counting (Fiji v.2.0.0) and StarDist Fiji plugin (4).

Immunofluorescence in iNeurons

For MAP2B quantification, induced neurons were differentiated in glass coverslips until day 10, 20 and 30. iNeurons were fixed in 4% paraformaldehyde in PBS for 15 min at room temperature immediately after removal of the culture medium. iNeurons were then washed 3 times for 5 min with PBS, permeabilized with 0.1% Triton X-100 in PBS for 15 min, and blocked in 5% donkey serum in PBS for 30 min. After blocking, the cells were incubated with primary antibodies diluted in a blocking solution overnight at + 4°C. The cells were washed three times with PBS for 5 min and incubated with secondary antibodies at room temperature

for 1 h. Neuronal nuclei were then stained with DAPI solution at room temperature for 5 min. Coverslips were rinsed in sterile water and mounted on a glass slide with 5 µl of Mowiol mounting medium. Primary antibodies used: MAP2B 1:500 (BD Biosciences 610460). Images were acquired with Leica SP8 confocal microscope and processed with Fiji (v. 2.0.0).

Western Blot

Proteins were extracted using SDS buffer (4.8% SDS, 20% glycerol, 0.1 M Tris-HCl, pH 7.5). Cell pellets were lysed with 5 volumes of boiling SDS buffer, denaturated for 10 min at 95°C and sonicated. Protein lysates were centrifuged for 10 min at 13000 rpm, RT and supernatants were quantified using BCA (Pierce BCA Protein assay kit, 23225). Proteins were resolved by and blotted on the Transfer membrane (Immobilon-P, Merck Millipore IPVH00010). Membrane blocking (5% BSA/TBS, 0.2% Tween-20 for 1h) was followed by incubation with the specific primary antibody and HRP-conjugated secondary antibodies. Proteins were detected by ECL (Bio-Rad). Antibodies used in the study: anti-VCL (Sigma, V9131-2ML; 1:20000); anti-GTF2I (BD Biosciences 610942; 1:3000); anti-BAZ1B (Abcam ab50632; 1:1000); anti-NSUN5 (Abcam ab121633; 1:1000); anti-TBL2 (Abcam ab171297; 1:1000); anti-EIF4H (Cell Signaling 2444S; 1:1000); anti-4EBP (Cell Signaling 9644; 1:1000); anti-p4EBP1 (Cell Signaling 9451 (Ser65); 1:1000); anti-p4EBP1 (Cell Signaling 2855S (Thr37/46); 1:1000); anti-pRPS6 240/244 (Cell Signaling 5364S; 1:1000); anti-pRPS6 Ser235/236 (D57.2.2E; Cell-signaling 4858S); anti-tot RPS6 (Cell Signaling 2317); anti-puromycin (Millipore MABE343; 1:1000); anti-GAPDH (Millipore ABS16; 1:10000); anti-EIF2alpha (Cell Signaling 33985; 1:1000); anti-p-eIF2a (S51) (D9G8) Cell Signaling 3398S) and anti-REST (Millipore 07-579;1:1000); anti-TUBULIN β-3 (TUBB3) (BioLegend, 802001; 1:30000). Quantification was done with ImageJ, while Prism 9 was used for the statistical analysis.

Puromycin incorporation assay

The Puromycin incorporation assay was done as previously described (5). Briefly, iNeurons were treated for 20 min with 100 µg/ml of puromycin, lysed and profile by western blotting as described above.

RNA extraction, RT-PCR and qPCR

RNA was isolated using the RNeasy mini-Kit (QIAGEN) and genomic DNA was removed using the RNase-Free DNase Set (QIAGEN). Retrotranscribed cDNA was obtained from 1 µg of total RNA using the SuperScript VILO RetroTranscription kit from Life Technologies according to the manufacturer's instructions.

For RT-qPCR analysis, a total cDNA amount corresponding to 5 ng of starting RNA was used for each reaction. TaqMan Fast Advanced Master Mix from Life Technologies and 900 nM TaqMan Gene Expression Assay for each gene analyzed were used in a 10 µl volume reaction. A QuantStudio 6 Flex Real-Time PCR system (Applied Biosystems) was utilized to determine the Ct values. Relative mRNA expression levels were normalized to housekeeping genes and analyzed through the comparative delta-delta Ct method using the QBase Biogazelle software.

RNA-seq libraries preparation

Library preparation for RNA-seq was done with the Ribo-Zero Total RNA sample preparation kit (Illumina), starting from 250 ng to 1 g of total RNA. The quality of cDNA libraries was assessed by Agilent 2100 Bioanalyzer using the High Sensitivity DNA Kit. Libraries were sequenced with the Illumina HiSeq (main experiments) or NovaSeq (REST experiment), 50–base pair (bp) paired-end with a coverage of 35 million reads per sample.

Ribosomal profiling

Libraries of ribosome-protected fragments (RPFs) were generated using the TruSeq Ribo Profile (Mammalian; Illumina). The experiment was done in three biological replicates for each genotype. Briefly, iNeurons were treated with cycloheximide at 0.1mg/mL for 5 min and lysed according to the manufacturer's instructions. To generate Ribosome Footprints, cell lysates were digested with TruSeq Ribo Profile Nuclease. The ribosome-protected fragments were first purified using MicroSpin S-400 columns (GE) and then size-selected (28-30 nt) from 15% polyacrylamide/7-8 M urea/TBE gel. rRNA was removed using the Ribo-Zero Gold Kit (Illumina). After 3' adaptor ligation and reverse transcription of the library, cDNA (70-80 nt) was purified from 10% polyacrylamide/7-8 M urea/TBE gel. After cDNA circularization and limited amplification (9 cycles), the RPF libraries were purified using an 8% native polyacrylamide gel. The RPF libraries were sequenced on an Illumina HiSeq 2500 sequencer with a single-end 50 cycles (SE50) run type.

MS-based Proteomics

Cell pellets from isogenic lines were processed and digested using the PreOmics iST sample preparation kit, following the manufacturer's guidelines. Peptide mixtures were separated by reversed-phase chromatography on an EASY-nLC 1200 ultra-high-performance liquid chromatography (UHPLC) system through an EASY-Spray column (Thermo Fisher Scientific), 25-cm long (inner diameter 75 µm, PepMap C18, 2 µm particles), which was connected online to a Q Exactive HF (Thermo Fisher Scientific) instrument through an EASY-Spray™ Ion Source (Thermo Fisher Scientific). Peptides were loaded in buffer A (0.1% formic acid) and eluted with a linear 135 min gradient of 3–30% of buffer B (0.1% formic acid, 80% (v/v) acetonitrile), followed by a 10 min increase to 40% of buffer B, 5 min to 60% buffer B and 2 min to 95% buffer B, at a constant flow rate of 250 nl/min. The column temperature was

kept at 45°C under EASY-Spray oven control. The mass spectrometer was operated in a top-10 data-dependent acquisition (DDA) mode. MS spectra were collected in the Orbitrap mass analyzer at a 60,000 resolution (200 m/z) within a range of 375–1650 m/z with an automatic gain control target of 3e6 and a maximum ion injection time of 20 ms. The 10 most intense ions from the full scan were sequentially fragmented with an isolation width of 2 m/z, following higher-energy collisional dissociation with a normalized collision energy of 28%. The resolution used for MS/MS spectra collection in the Orbitrap was 15,000 at 200 m/z with an AGC target of 1e5 and a maximum ion injection time of 80 ms. Precursor dynamic exclusion was enabled with a duration of 20s. Each sample was injected twice, to increase protein identifications.

The MS data were processed with MaxQuant (6), using the Uniprot HUMAN(181029) database. Enzyme specificity was set to trypsin and two missed cleavages were allowed. Methionine oxidation and N-terminal acetylation were included as variable modifications and the FDR was set to 1%, both at the protein and peptide level. The label-free software MaxLFQ (54) was activated, as well as the "match between runs" feature (match from and to, matching time window=2 min). The mass spectrometry data have been deposited to the ProteomeXchange Consortium (7) via the PRIDE partner repository with the dataset identifier PXD035276.

For patient-derived cell series, a published, fast, quantitative SWATH-MS method was deployed (8, 9). Briefly, the SCIEX 5600 TripleTOF instrument was specifically tuned to perform the 64-variable window SWATH-MS using a 60-min LC-gradient was described previously (10). The SCIEX 5600 TripleTOF was interfaced with an Eksigent NanoLC Ultra 1Dplus HPLC system. Peptides were directly injected onto a 20-cm PicoFrit emitter (New Objective, self-packed to 20 cm with Magic C18 AQ 3-μm 200-Å material), and then separated using a 60-min gradient from 5–35% (buffer A 0.1% (v/v) formic acid, 2% (v/v) acetonitrile,

buffer B 0.1% (v/v) formic acid, 100% (v/v) acetonitrile) at a flow rate of 300 nL/min. The effective SWATH-MS windows include 399.5~408.2, 407.2~415.8, 414.8~422.7, 421.7~429.7, 428.7~437.3, 436.3~444.8, 443.8~451.7, 450.7~458.7, 457.7~466.7, 465.7~473.4, 472.4~478.3, 477.3~485.4, 484.4~491.2, 490.2~497.7, 496.7~504.3, 503.3~511.2, 510.2~518.2, 517.2~525.3, 524.3~533.3, 532.3~540.3, 539.3~546.8, 545.8~554.5, 553.5~561.8, 560.8~568.3, 567.3~575.7, 574.7~582.3, 581.3~588.8, 587.8~595.8, 594.8~601.8, 600.8~608.9, 607.9~616.9, 615.9~624.8, 623.8~632.2, 631.2~640.8, 639.8~647.9, 646.9~654.8, 653.8~661.5, 660.5~670.3, 669.3~678.8, 677.8~687.8, 686.8~696.9, 695.9~706.9, 705.9~715.9, 714.9~726.2, 725.2~737.4, 736.4~746.6, 745.6~757.5, 756.5~767.9, 766.9~779.5, 778.5~792.9, 791.9~807, 806~820, 819~834.2, 833.2~849.4, 848.4~866, 865~884.4, 883.4~899.9, 898.9~919, 918~942.1, 941.1~971.6, 970.6~1006, 1005~1053, 1052~1110.6, 1109.6~1200.5 (containing 1 m/z for the window overlap). SWATH MS2 spectra were collected from 50 to 2,000 m/z. The collision energy (CE) was optimized for each window according to the calculation for a charge 2+ ion centered upon the window with a spread of 15 eV. An accumulation time (dwell time) of 50 ms was used for all fragment-ion scans in high-sensitivity mode. To detect proteins from SWATH-MS dataset, a published data extraction framework (10), including establishing SWATH assay library from 150-min shotgun analysis on the measured neuron cells, wiff files format conversion (to mzXML files), OpenSWATH [PMID: 24727770] based FDR control (Targeted peptide-FDR 1%), and TRIC (TRansfer of Identification Confidence) alignment (11) was followed. To quantify the protein abundance levels across samples, the requantification feature in OpenSWATH was enabled for the filtered protein list. We summed up the most abundant peptides for each protein (i.e., the top 3 peptide groups based on intensity were used for those proteins identified with more than three proteotypic peptide signals whereas all the peptides were summarized for other proteins).

Electrophysiological recordings

Patient-derived neurons were differentiated until day 35. Intrinsic neuronal excitability was measured using whole-cell patch-clamp recording in the current-clamp configuration by injecting 500 msec 5 pA current steps from -60 mV as previously described (12). Patch-clamp recordings were performed in an extracellular solution with the following composition: 130 mM NaCl, 5 mM KCl, 1.2 mM KH2PO4, 1.2 mM MgSO4, 2 mM CaCl2, 25 mM HEPES, and 6 mM Glucose, pH 7.4, in the presence of synaptic transmission blockers, CNQX 10 mM and APV 20 mM. Borosilicate glass pipettes of 4-6 MΩ were filled with the following internal solution (in mM): 135 K-gluconate, 5 KCl, MgCl2, 10 HEPES, 1 EGTA, 2 ATP, 0.5 GTP, pH 7.4. Electrical signals were amplified by a Multiclamp 200 B (Axon instruments), filtered at 5 kHz, digitized at 20 kHz with a digidata 1440 and stored with pClamp 10 (Axon instruments). Passive properties including capacitance and membrane resistance were calculated in voltage-clamp mode using a pulse test of 10 mV. Only neurons with a stable (max deviation 10%) access resistance <15 MΩ and with a holding current < 100 pA were considered. Intrinsic neuronal excitability was calculated as the total number of action potentials for each current step, whereas the current threshold density was calculated as the minimum depolarizing current needed to elicit at least one action potential. The parameters describing the action potential shape (AP peak and Max Rising Slope) were analyzed using the pClamp software (Molecular Devices) and data were analyzed using Prism software (GraphPad Software, Inc.).

Sodium (I_{Na}) and potassium (I_k) currents were recorded in voltage-clamp mode using a step protocol from -120 mV to + 50 mV at 10 mV increments from a holding potential of -70 mV. Only neurons with a stable access resistance < 5MΩ (uncompensated) were considered.

For the experiments with REST, inhibitor X5050 (Millipore) was dissolved in DMSO and 100uM was added in the neuronal medium for 24 hours before recordings or harvesting neurons for RNA extraction and RNA sequencing.

Transcriptome-based staging

Since staging against a reference dataset requires relative rather than absolute transcriptome similarity and technical differences between profiling methods can often distort similarity estimates, we employed a correction method before assessing similarity. For each gene, the minimum distance between the (edgeR-normalized) logCPM of any sample of the query dataset and that of any sample of the reference dataset was removed from the query dataset before similarity calculation. Similarity was then calculated using Pearson correlation.

For the reference datasets, we used the original quantification and clustering from the respective authors. For comparison to Lin et al. Ngn2 dataset (13), we downloaded the single-cell expression and annotation data from ArrayExpress, and excluded clusters 5 and 8 as astrocytes, as well as cells in time points other than the first from clusters displaying high expression of pluripotency genes. Pseudo-bulk profiles were then created by summing the counts of remaining cells from each timepoint, and similarity was calculated as described above.

References:

1. Fabris S, Agnelli L, Mattioli M, Baldini L, Ronchetti D, Morabito F, et al. Characterization of oncogene dysregulation in multiple myeloma by combined FISH and DNA microarray analyses. Genes Chromosomes Cancer. 2005;42(2):117-27.
2. Pasca AM, Sloan SA, Clarke LE, Tian Y, Makinson CD, Huber N, et al. Functional cortical neurons and astrocytes from human pluripotent stem cells in 3D culture. Nat Methods. 2015;12(7):671-8.
3. Lopez-Tobon A, Shyti R, Villa CE, Cheroni C, Fuentes-Bravo P, Trattaro S, et al. GTF2I dosage regulates neuronal differentiation and social behavior in 7q11.23 neurodevelopmental disorders. Sci Adv. 2023;9(48):eadh2726.
4. Uwe Schmidt MW, Coleman Broaddus, Gene Myers. Cell Detection with Star-convex Polygons. arXiv:180603535 [csCV]. 2018.
5. Cox J, Hein MY, Luber CA, Paron I, Nagaraj N, Mann M. Accurate proteome-wide label-free quantification by delayed normalization and maximal peptide ratio extraction, termed MaxLFQ. Mol Cell Proteomics. 2014;13(9):2513-26.
6. Tyanova S, Temu T, Cox J. The MaxQuant computational platform for mass spectrometry-based shotgun proteomics. Nat Protoc. 2016;11(12):2301-19.
7. Vizcaino JA, Deutsch EW, Wang R, Csordas A, Reisinger F, Rios D, et al. ProteomeXchange provides globally coordinated proteomics data submission and dissemination. Nat Biotechnol. 2014;32(3):223-6.
8. Collins BC, Hunter CL, Liu Y, Schilling B, Rosenberger G, Bader SL, et al. Multi-laboratory assessment of reproducibility, qualitative and quantitative performance of SWATH-mass spectrometry. Nat Commun. 2017;8(1):291.
9. Gillet LC, Navarro P, Tate S, Rost H, Selevsek N, Reiter L, et al. Targeted data extraction of the MS/MS spectra generated by data-independent acquisition: a new concept for consistent and accurate proteome analysis. Mol Cell Proteomics. 2012;11(6):O111 016717.
10. Liu Y, Mi Y, Mueller T, Kreibich S, Williams EG, Van Drogen A, et al. Multi-omic measurements of heterogeneity in HeLa cells across laboratories. Nat Biotechnol. 2019;37(3):314-22.
11. Rost HL, Liu Y, D'Agostino G, Zanella M, Navarro P, Rosenberger G, et al. TRIC: an automated alignment strategy for reproducible protein quantification in targeted proteomics. Nat Methods. 2016;13(9):777-83.
12. Pozzi D, Lignani G, Ferrea E, Contestabile A, Paonessa F, D'Alessandro R, et al. REST/NRSF-mediated intrinsic homeostasis protects neuronal networks from hyperexcitability. EMBO J. 2013;32(22):2994-3007.
13. Lin HC, He Z, Ebert S, Schornig M, Santel M, Nikolova MT, et al. NGN2 induces diverse neuron types from human pluripotency. Stem Cell Reports. 2021;16(9):2118-27.

Supplementary Figures and Legends

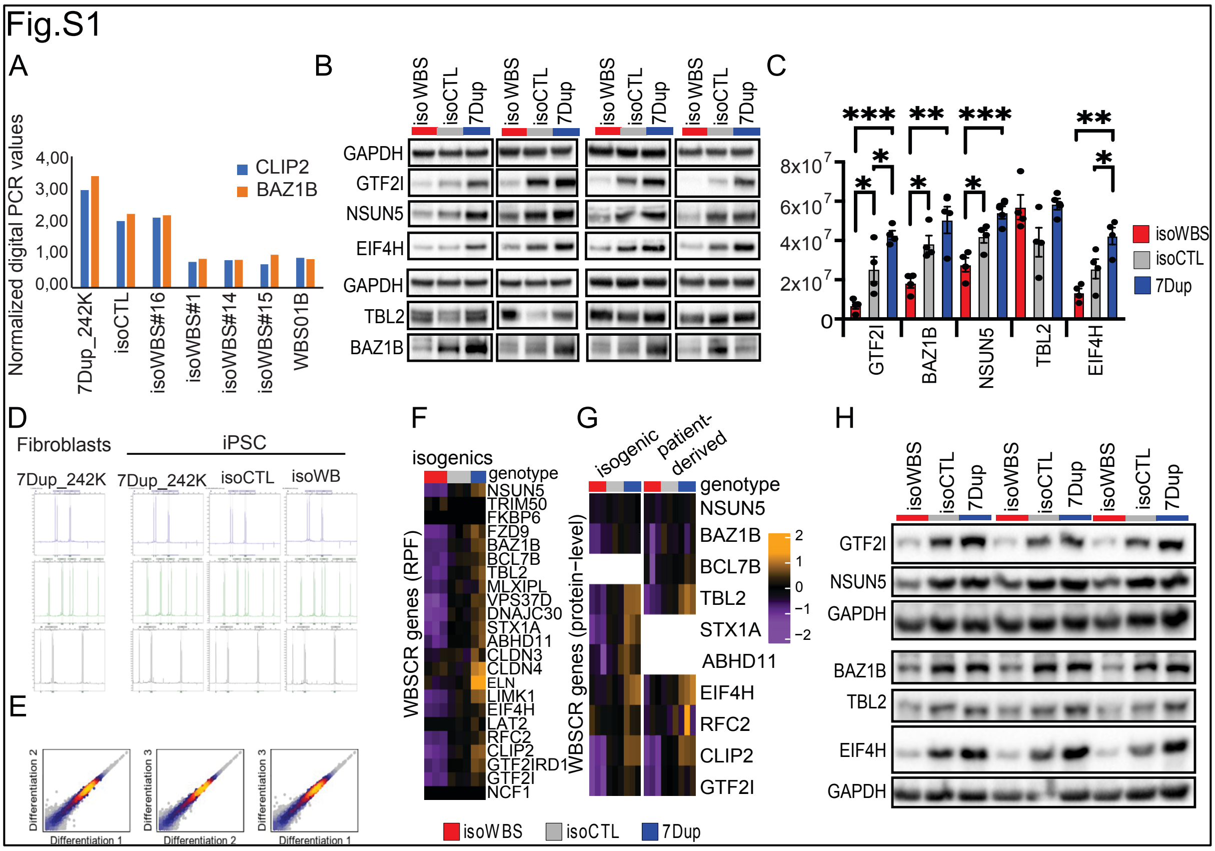

Figure S1 7q11.23 isogenic iPSC and iNeurons preserve dosage. (A) Digital PCR validation of isogenic lines. The analysis was done on DNA from the original patient-derived 7Dup line (242K), isoCTL, several isoWBS clones and one patient-derived WBS line (01B) as a positive control. The probes were designed for two genes from WBSCR, CLIP2 and BAZ1B. **(B-C)** Western blot analysis **(B)** and quantification **(C)** for several WBSCR genes in iPSC showed that the dosage is maintained at the protein level. GAPDH was used as a loading control. Not-normalized data are shown as mean ± SEM (n=4). The statistical comparisons were done with one-way ANOVA followed by Tukey's multiple comparison test. The significance level was set at *p<0.05; **p<0.01; ***p<0.001; ****p<0.0001; isoCTL - isogenic control; isoWBS - isogenic WBS line. **(D)** Short tandem repeats (STR) analysis confirmed the identity of generated isogenic lines. In addition to the original iPSC 7Dup patient line (242K), we also used the fibroblast from the patient as a control. **(E)** The PiggyBac system allows for excellent reproducibility across different rounds of neuronal differentiation (mean logCPM correlation of 0.997). **(F-G)** Relative expression of WBSCR genes in iNeurons, at the level of Ribosome-protected fragments (RPF; **F**) and protein **(G)**. All detected genes are shown in genomic order. **(H)** Biological replicates of western blot analysis of WBSCR genes is shown in Figure 1D, quantification of which is shown in Figure 1E.

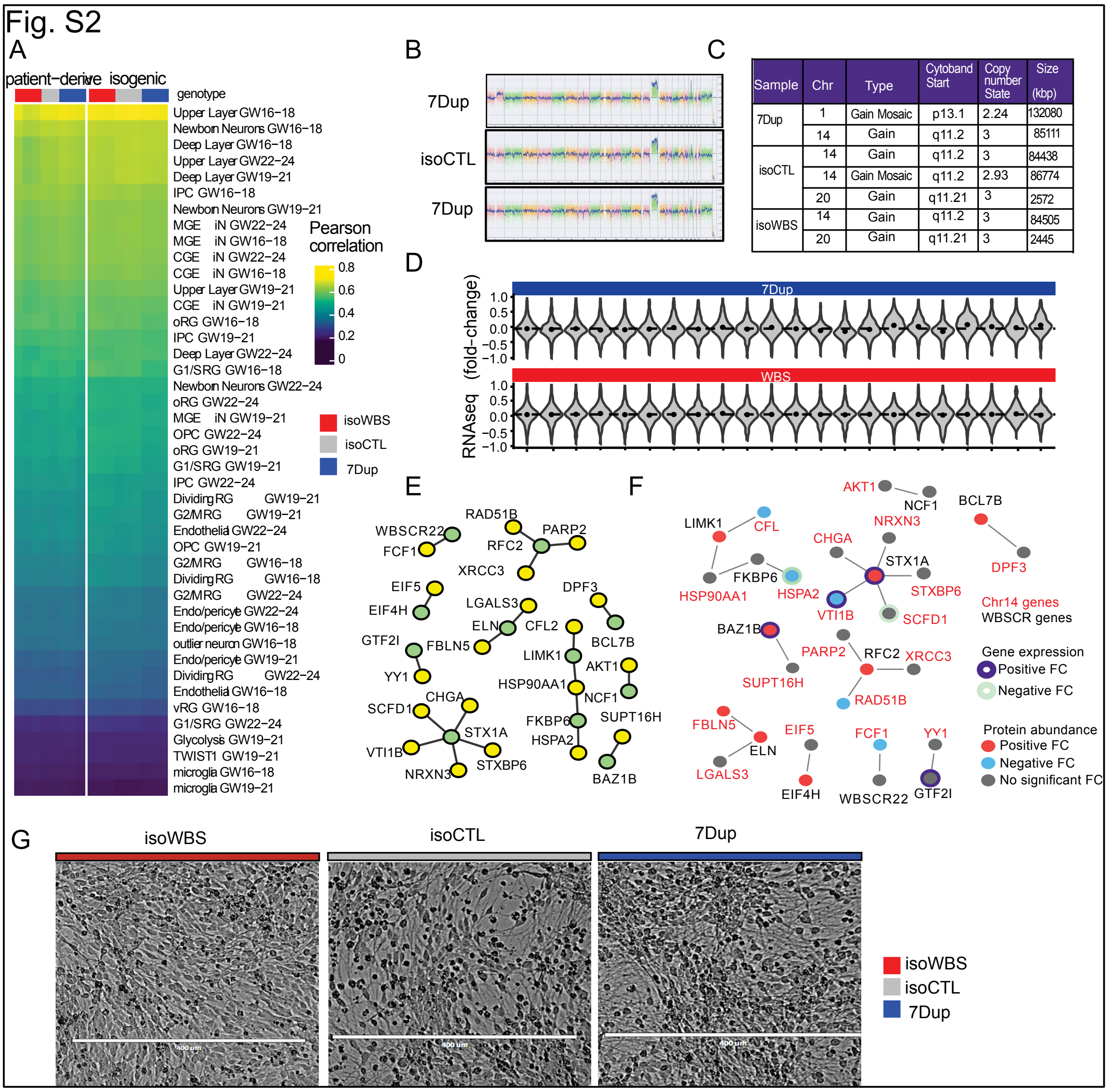

Sample	Chr	Type	Cytoband Start	Copy number State	Size (kbp)
7Dup	1	Gain Mosaic	p13.1	2.24	132080
	14	Gain	q11.2	3	85111
isoCTL	14	Gain	q11.2	3	84438
	14	Gain Mosaic	q11.2	2.93	86774
	20	Gain	q11.21	3	2572
isoWBS	14	Gain	q11.2	3	84505
	20	Gain	q11.21	3	2445

Figure S2 7q11.23 isogenic lines contain a large duplication on Chr14. (A) Correlation of iNeurons log(CPM) values with cluster averages from scRNA-seq of human fetal neocortex development (1), after correcting for technical differences. **(B)** Karyostat analysis of DNA isolated from 30 days old iNeurons from the isogenic lines shows duplication of Chr14 in all three genotypes. **(C)** The table with the summary of genomic alterations identified in the karyostat analysis. **(D)** Fold-change distributions by chromosome in 7Dup vs isoCTL and isoWBS vs isoCTL do not show appreciable differences in the expression of Chr14. **(E)** Protein-protein interaction network between the WBSCR genes and genes from the duplicated region of Chr14 genes (based on the STRING (v11) high-confidence interactions defined as combined_score >= 0.7 and STRING experimental score >= 0.1). Chr14 proteins are in yellow, and WBSCR proteins are in green. **(F)** Overlay of differential gene expression and differential protein abundance between isogenic line vs 7Dup. Only fold changes with FDR equal to or smaller than 0.05 are shown. Labels in red represent Chr14 genes, node color represents FC (red: positive FC, blue: negative FC, grey: no significant FC) and node border represents protein abundance FC (purple: positive FC, green: negative FC, no border: no significant FC). **(G)** Brightfield images of isogenic iNeurons on day 14. While isoWBS iNeurons show round cell bodies, isoCTL and 7Dup already have thinner and elongated neurites.

Fig. S3

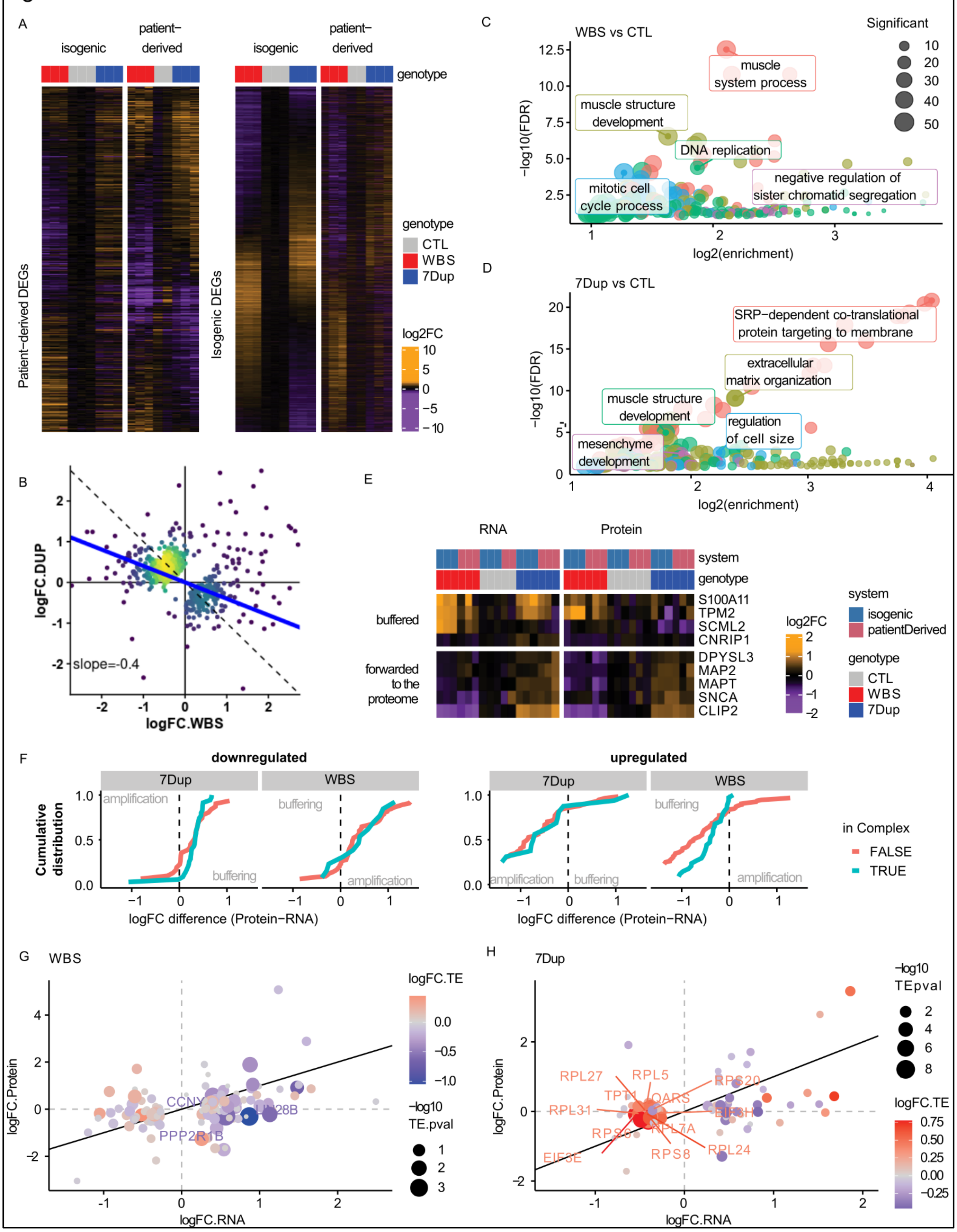

Figure S3 Differential translation of distinct classes of genes in WBS and 7Dup. (A) Heatmaps showing the fold-changes, in each system, of the DEGs identified in patient-derived lines (left) or isogenic lines (right) alone. **(B)** Correlation between RNA foldchanges in WBS and 7Dup (vs CTL) of the union of genes found significant in either comparison. **(C-D)** Enriched Gene Ontology terms in the WBS vs CTL **(C)** and 7Dup vs CTL **(D)**. Similar terms are clustered (denoted by colors) and only the top term per cluster is shown. **(E)** Example genes whose differential expression at the RNA level is either buffered or forwarded to the proteome.

(F) Cumulative distribution plots of the differences between RNA and Protein log-fold changes split by genotype and direction of the transcriptomic change. The fact that most of the distribution shows a positive difference for downregulated genes and a negative one for upregulated genes is consistent with an overall mild buffering. Genes known to form complexes, which have been reported in other contexts to undergo buffering through complex-mediated stabilization and excess degradation, showed only a slightly stronger effect, especially among genes downregulated in 7Dup or upregulated in WBS. **(G-H)** Differential translation efficiency (TE) of specific genes in WBS **(G)** and 7Dup **(H)**, using the integrated analysis across the three layers. The labeled genes are those with a significant TE effect.

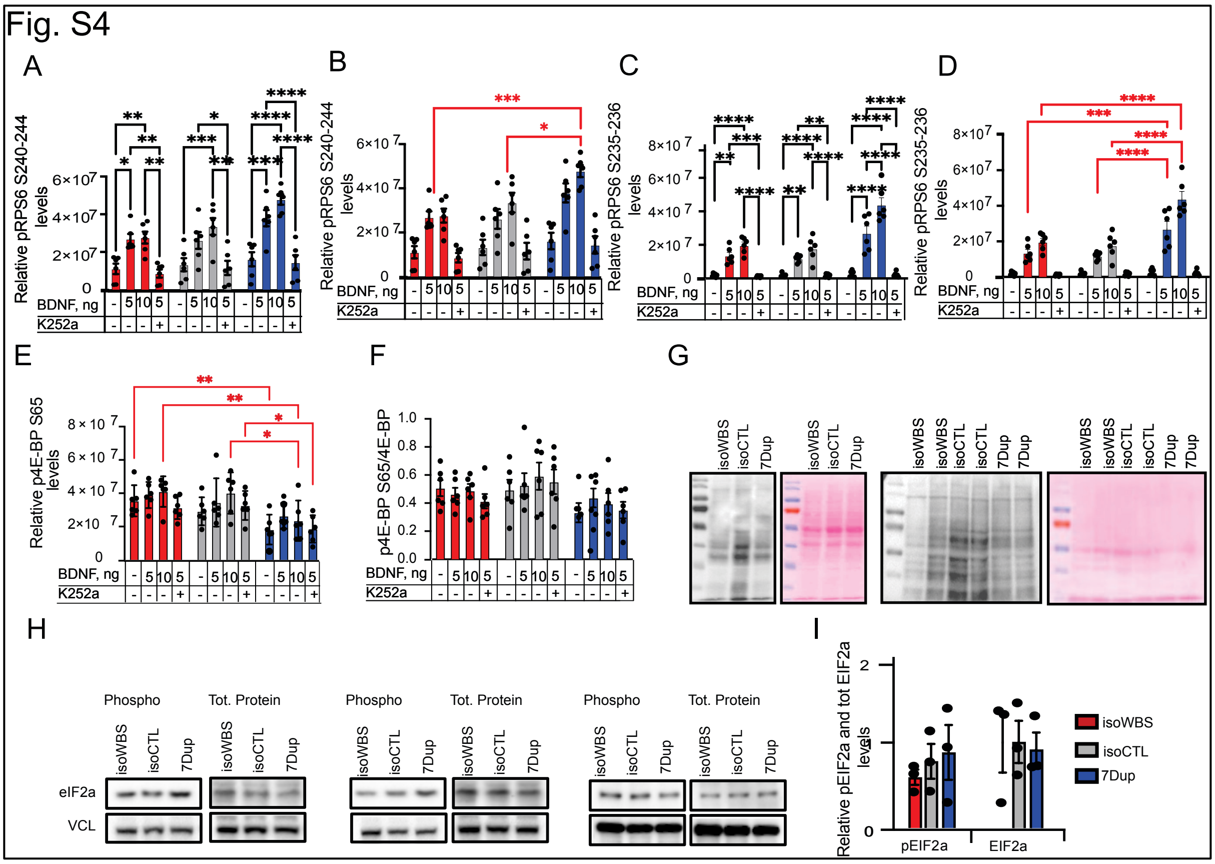

Figure S4 Phosphorylation of EIF2α does not change significantly between genotypes. Quantification of pRPS6 S240-244 **(A-B)**. The same quantification is shown in both graphs with the black asterisks showing the significance between treatments **(A)** and red asterisks between genotypes **(B)**. pRPS6 S235-236 **(C-D)**. The same quantification is shown in both graphs with the black asterisks showing the significance between treatments **(C)** and red asterisks between genotypes **(D)**. p4EBP S65 **(E)**, and p4EBP S65 normalized to total levels of 4EBP **(F).** Quantified western blots are shown in **Figure 5H** and **S5**. The experiment was done in 6 replicates. **(G)** Western blots analysis and the corresponding ponceau of puromycin incorporation assays. Three independent experiments, done in two rounds of differentiation are shown. Quantification is shown in **Figure 4E. (H-I)** EIF2a phosphorylation does not change significantly between genotypes; western blots are shown in **(H)** and quantification in **(I)**. The results from three independent iNeuronal differentiations are shown. All data are shown as mean ± SEM. The statistical comparisons were done with two-way ANOVA followed by Tukey's multiple comparison tests for panels **A** and one-way ANOVA followed by Tukey's multiple comparison tests for panel **I**. The significance level was set at *p<0.05; **p<0.01; ***p<0.001; ****p<0.0001.

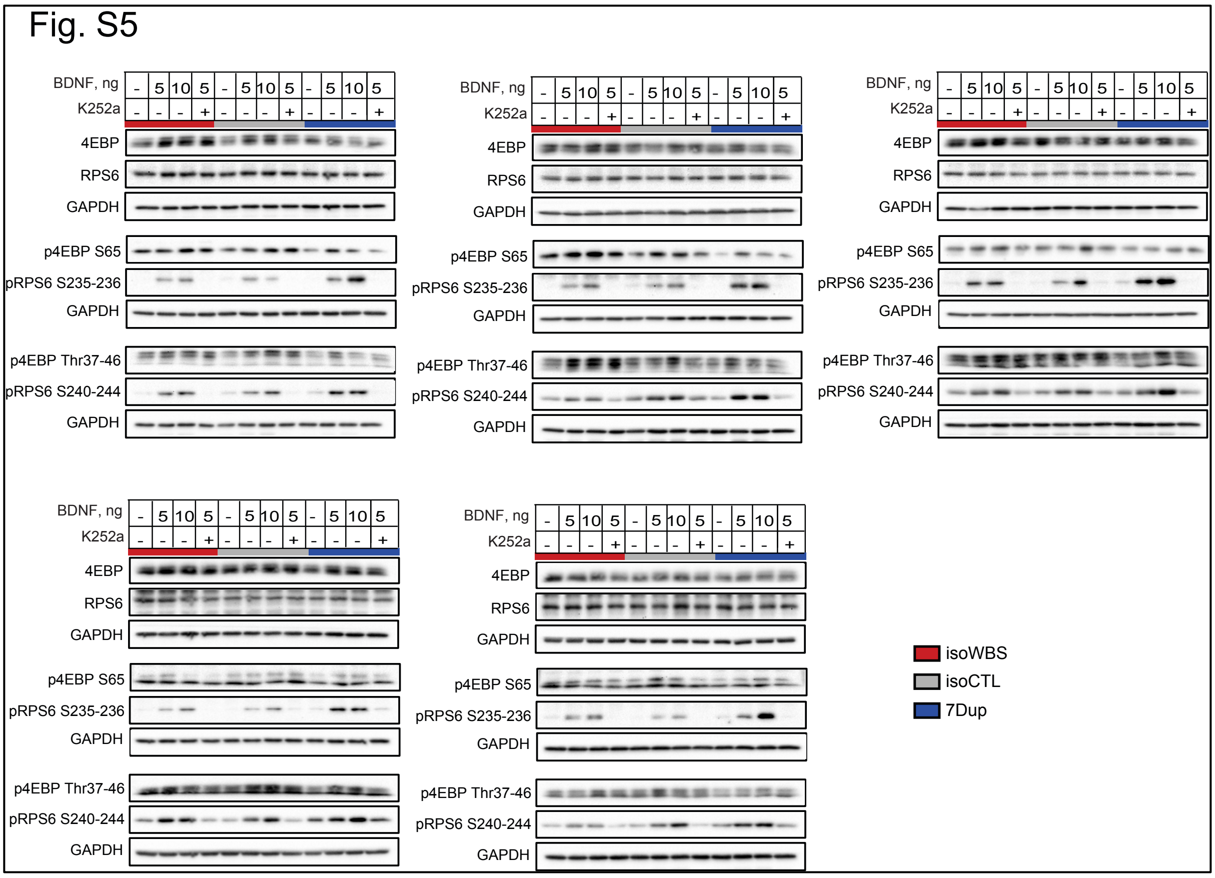

Figure S5 Western blot analysis of total RPS6, pRPS6 240-244, pRPS6 235-236, total 4EBP, p4EBP Thr37-47 and p4EBP S65 levels. The experiment was done with the extracts obtained from six different neuronal preparations.

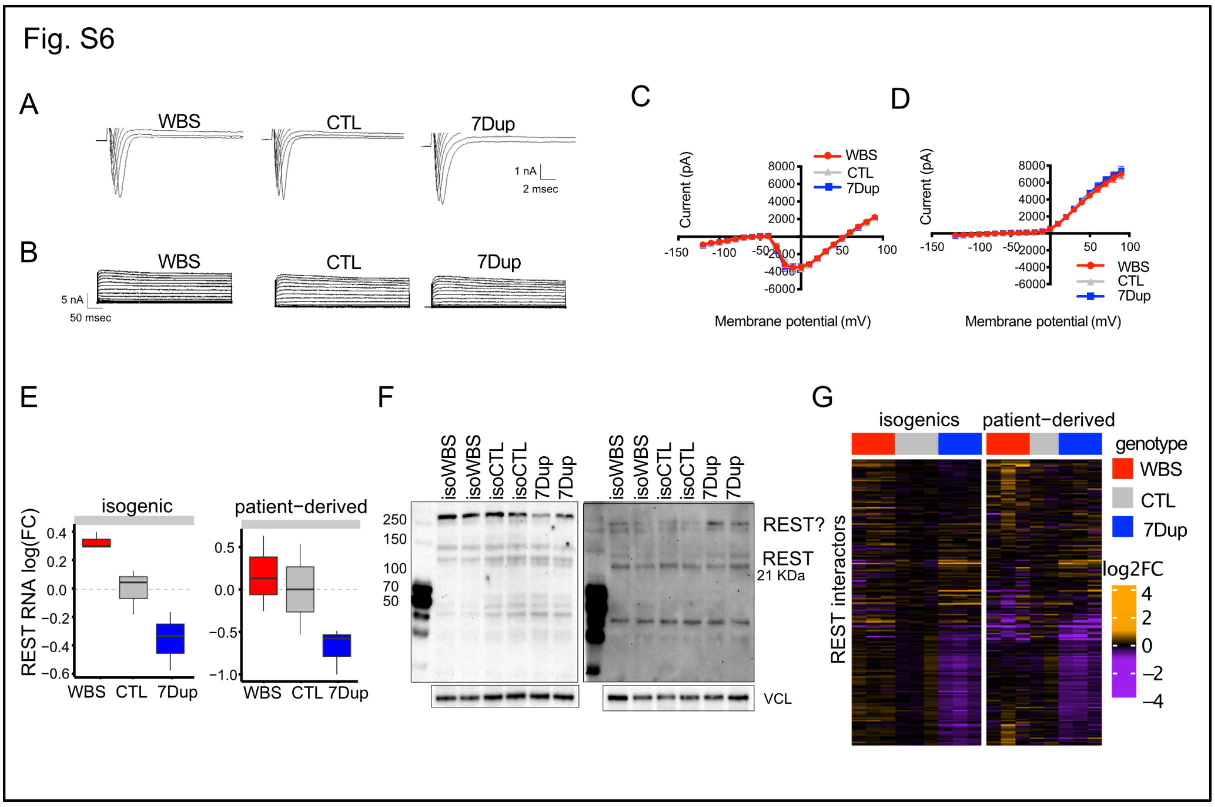

Figure S6 REST is regulated post-transcriptionally in WBS and 7Dup. (A-B) Representative sodium and potassium current traces in patient-derived iNeurons from the 3 genotypes were recorded in voltage-clamp mode using a step protocol from -120 mV to + 50 mV at 10 mV increments from a holding potential of -70 mV. **(C-D)** I-V plots of the sodium and potassium currents, respectively. WBS n=45 neurons, CTL n=41, 7Dup n=39. Data are shown as mean ± SEM. All the data is the average of 3 independent experiments. Comparisons were done using two-way ANOVA followed by Tukey's multiple comparison test. The significance level was set at *p<0.05; **p<0.01. **(E)** RNAseq analysis of REST mRNA expression in isogenic (left) and patient-derived iNeurons (right). **(F)** REST is buffered at the level of protein in all three genotypes. The predicted REST MW is 121 KDa, however, it is sometimes detected at 200 KDa. In iNeurons, we reproducibly detected bands above 250 KDa (marked with REST?), around 120 KDa (marked with REST), and around 40 KDa. One of the identified bands consistently changed between genotypes. Vinculin (VCL) was used as the loading control. **(G)** Heatmap showing changes in expression of the REST-interactome in a 7q11.23-dependent manner.

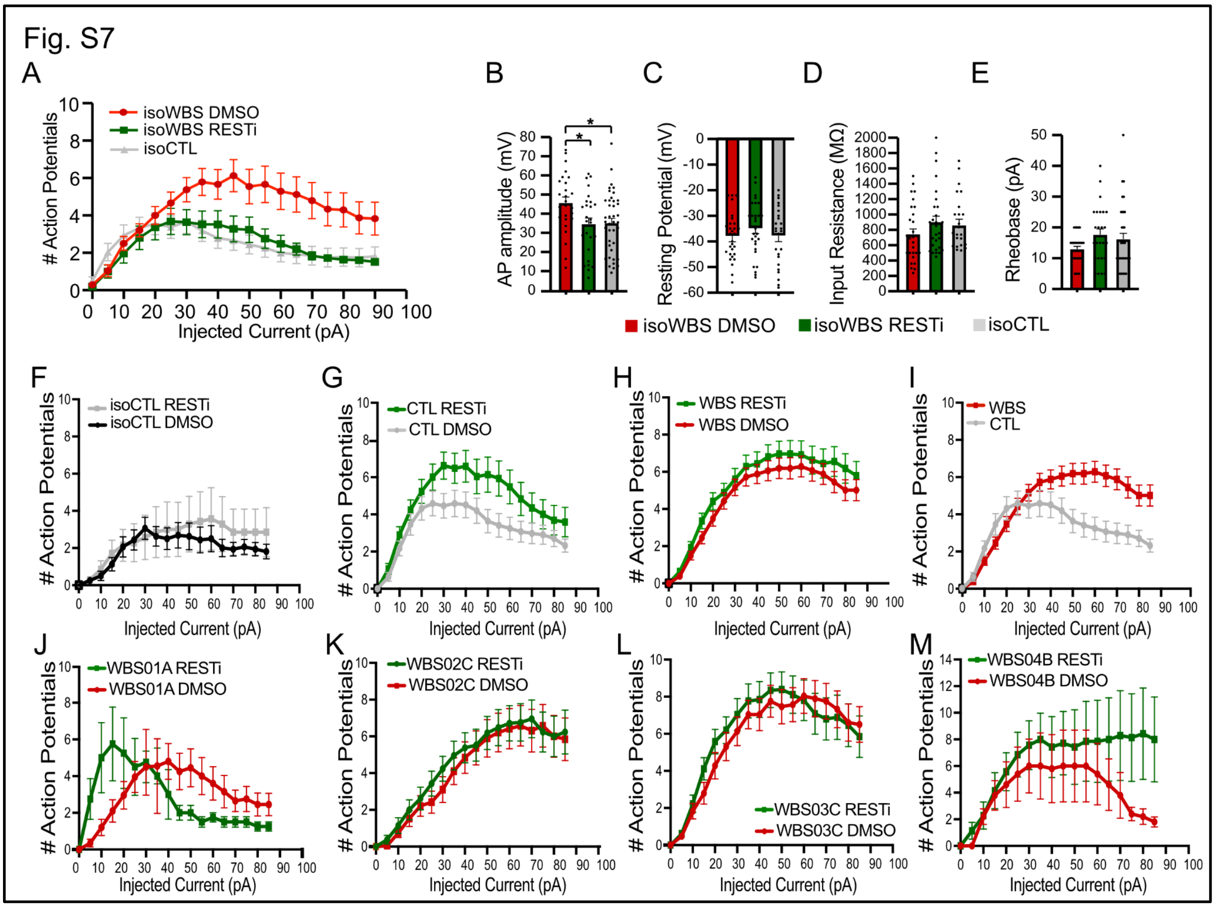

Figure S7. The effect of REST inhibition on isogenic and patient-derived CTL and WBS lines. (A) REST inhibition rescues AP frequency in isoWBS iNeurons. Quantitative analysis depicting the number of AP elicited in isoWBS iNeurons treated with RESTi or DMSO controls. Note how REST inhibition normalizes AP frequency to isoCTL iNeurons levels (isoCTLvs isoWBS DMSO, current step 45*; 50-55**; 60***; 65-75**; isoWBS DMSO vs isoWBS RESTi, 50-60*); isoWBS-DMSO, n=24 neurons; isoWBS-RESTi, n=25; isoCTL, n=27. **(B-E)** Passive properties were not affected by the REST inhibitor; AP amplitude instead was lowered. All the data is the average of 3 independent experiments. (**F**). REST inhibition does not affect AP frequency in isoCTL iNeurons (isoCTL DMSO=16 neurons; isoCTL RESTi=7 neurons). (**G**). REST inhibition does not affect AP frequency in CTL lines (CTL DMSO= 41 neurons; CTL RESTi= 29 neurons). (**H**). REST inhibition does not affect AP frequency in patient-derived WBS lines (WBS DMSO= 75 neurons; WBS RESTi=57 neurons). (**I**). Higher AP frequency in WBS patient-derived lines compared to CTL (current step 50-55**; 60-65***; 70-85**). Independent experiment in CTL (4 cell lines, n=41 neurons) and WBS (4 lines, n=75 neurons). (**J-M**). Data from WBS patient lines treated with RESTi or DMSO, and plotted independently. Data are shown as mean ± SEM. For comparing the data in (**A**), we used two-way ANOVA followed by Tukey's multiple comparison test, whereas for comparing passive properties we used one-way ANOVA followed by Tukey's multiple comparison test. For comparing data in (**F-I**), we used two-way ANOVA followed by Sidak's multiple comparison test. The significance level was set at $p<0.05$. *$p<0.05$; **$p<0.01$; ***$p<0.001$
