# Supplementary material for: Multiscale modeling uncovers 7q11.23 copy number variation–dependent changes in ribosomal biogenesis and neuronal maturation and excitability
Source: J Clin Invest. 2024 Jul 15;134(14):e168982. doi: 10.1172/JCI168982 (PMC11245157; doi:10.1172/JCI168982)

Full unedited blots related to Figure 1D and SF1H, differentiations #1, #2, #3 and #4

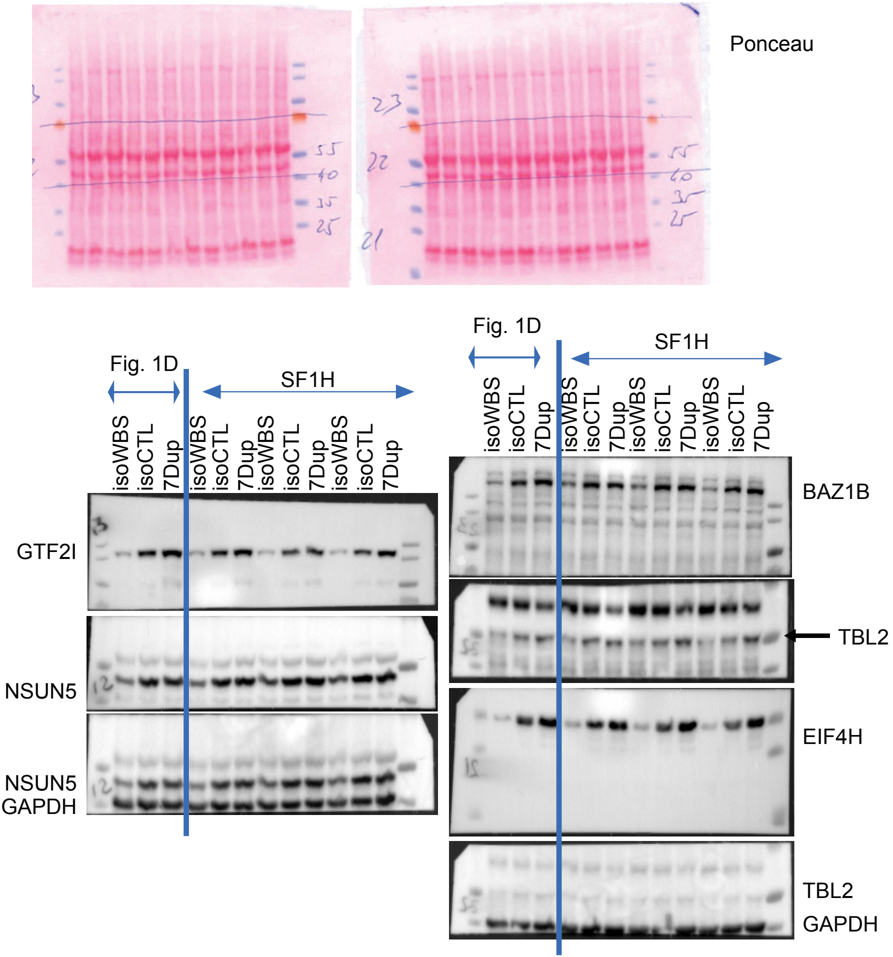

Full unedited blots for Figure S1B

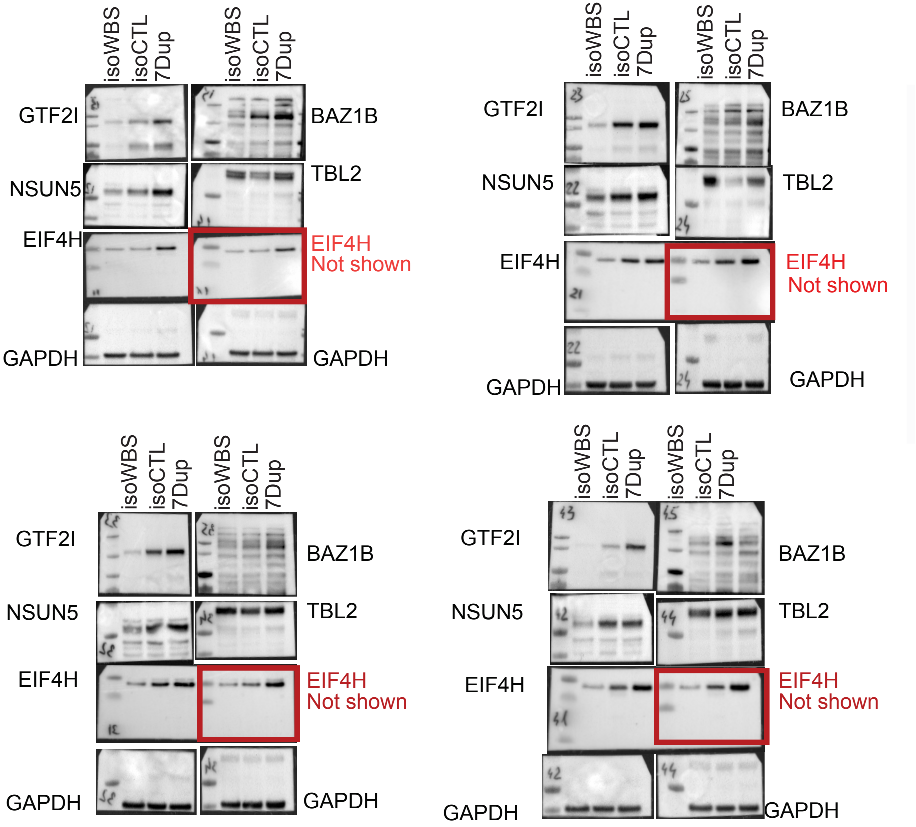

Full unedited blots related to Figure 5 and S5

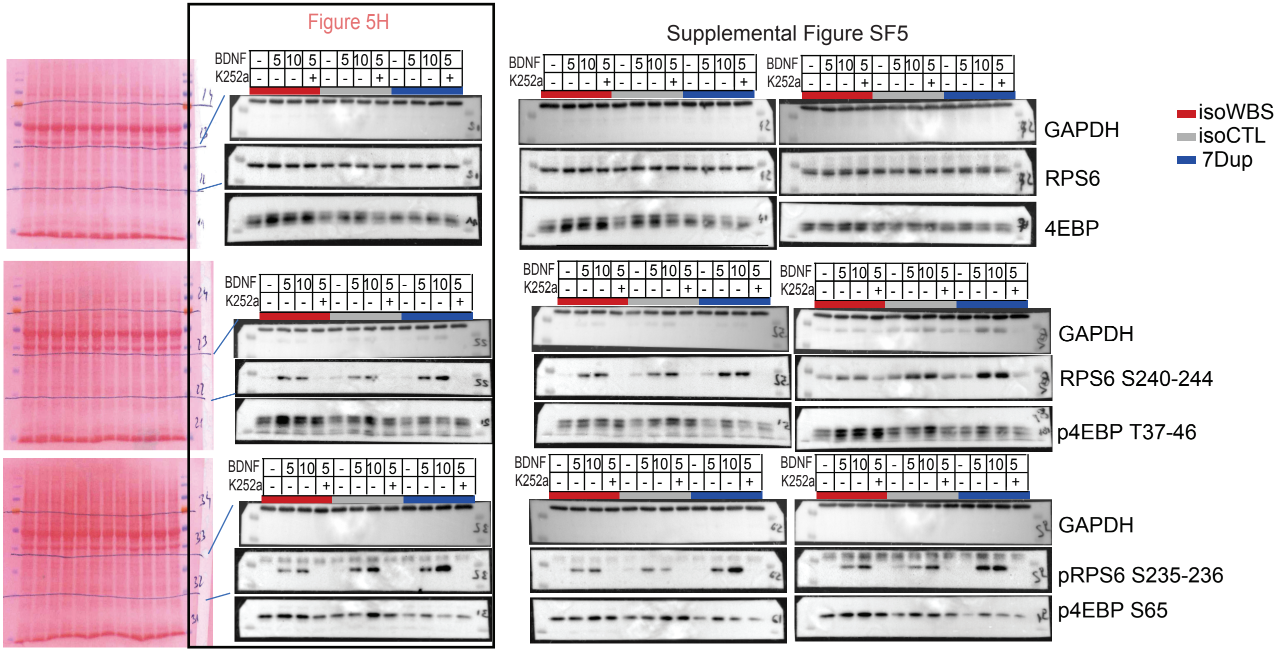

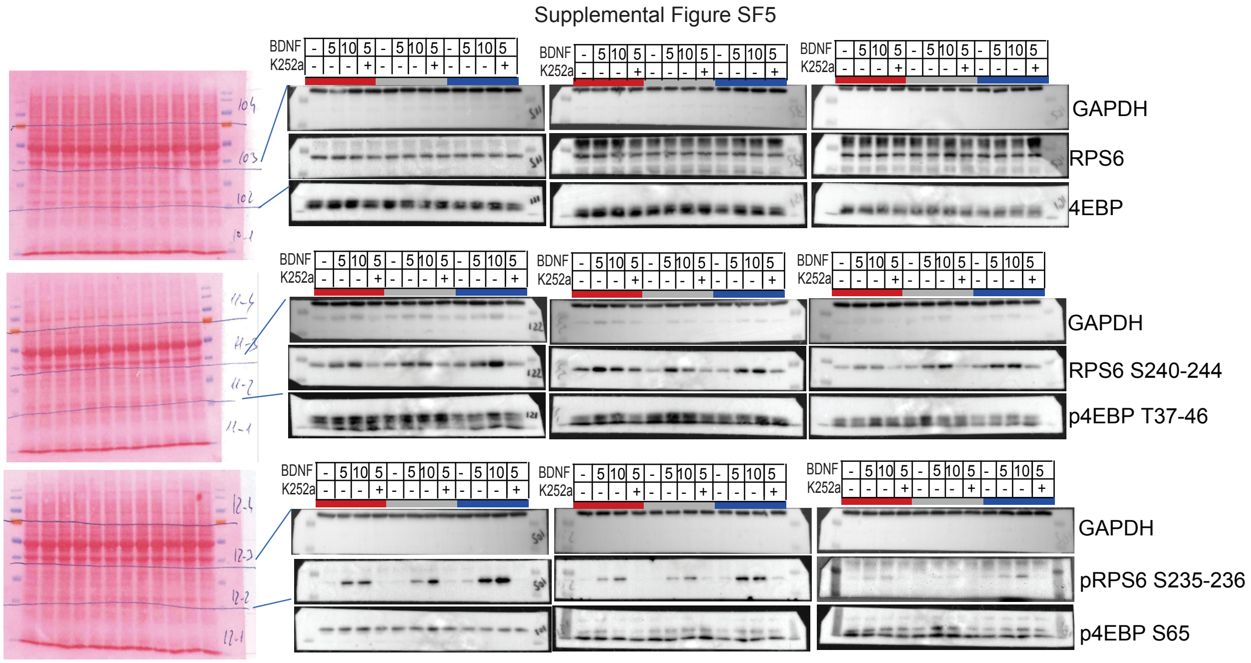

Full unedited blots related to Figures S4H. Only EIF2a, total and phospho, and vinculin (VCL) were used from these gels.

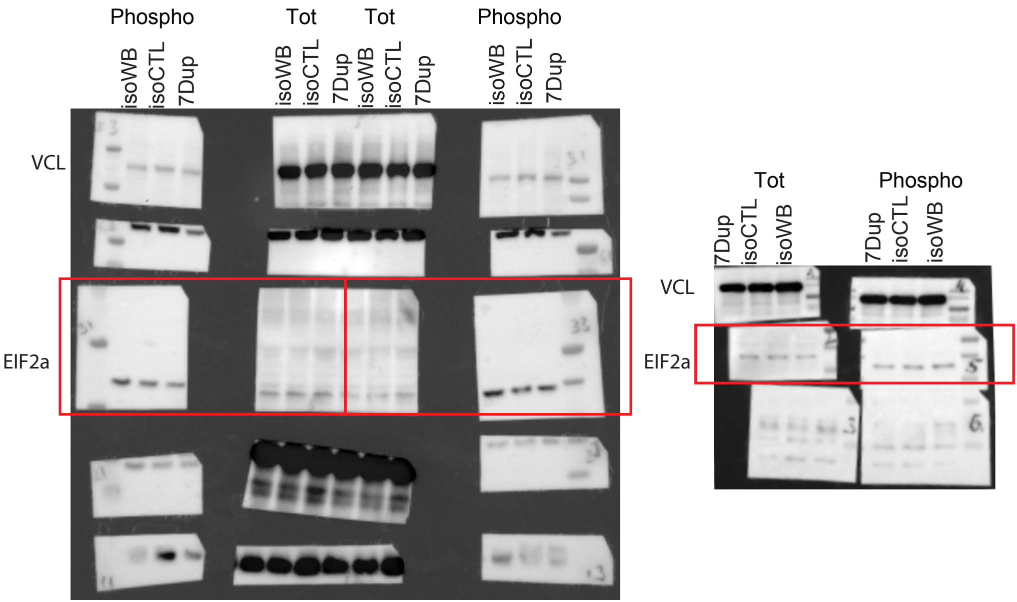

Full unedited blots related to Figure S6F

isoW BS
isoW BS
isoCTL
isoCTL
7Dup
7Dup

REST?
REST?

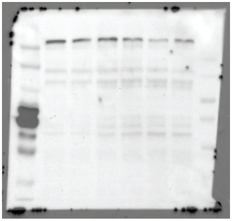

Second
incubation

isoW BS
isoW BS
isoCTL
isoCTL
7Dup
7Dup

VCL

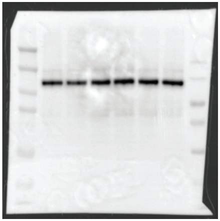

isoW BS
isoW BS
isoCTL
isoCTL
7Dup
7Dup

REST?
REST?

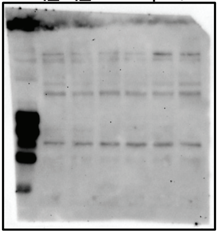

Second
incubation

isoW BS
isoW BS
isoCTL
isoCTL
7Dup
7Dup

VCL

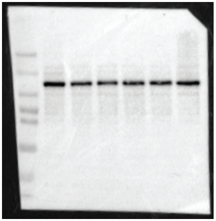

Full unedited blots related to Figures 5J and S4G

puromycin
incorporation
Figure 5J

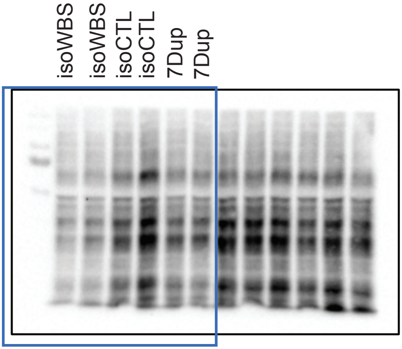

puromycin
incorporation
Figure S4G

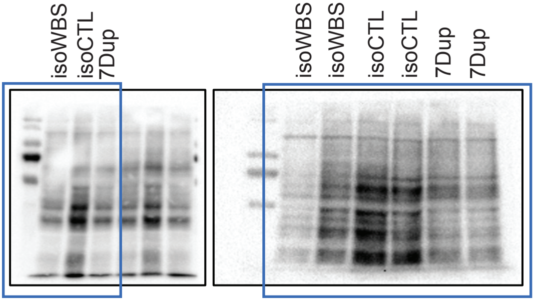

Supplement: Unedited blot and gel images [file jci-134-168982-s089.pdf]
